# Safety and infectivity of female cercariae in Schistosoma-naïve, healthy participants: a controlled human *Schistosoma mansoni* infection study

**DOI:** 10.1016/j.ebiom.2023.104832

**Published:** 2023-10-12

**Authors:** Jan Pieter R. Koopman, Emma L. Houlder, Jacqueline J. Janse, Miriam Casacuberta-Partal, Olivia A.C. Lamers, Jeroen C. Sijtsma, Claudia de Dood, Stan T. Hilt, Arifa Ozir-Fazalalikhan, Vincent P. Kuiper, Geert V.T. Roozen, Laura M. de Bes-Roeleveld, Yvonne C.M. Kruize, Linda J. Wammes, Hermelijn H. Smits, Lisette van Lieshout, Govert J. van Dam, Inge M. van Amerongen-Westra, Pauline Meij, Paul L.A.M. Corstjens, Simon P. Jochems, Angela van Diepen, Maria Yazdanbakhsh, Cornelis H. Hokke, Meta Roestenberg

**Affiliations:** a Leiden University Center for Infectious Diseases, Leiden University Medical Center, Albinusdreef 2, 2333 ZA, Leiden, the Netherlands; b Department of Cell and Chemical Biology, Leiden University Medical Center, Albinusdreef 2, 2333 ZA, Leiden, the Netherlands; c Center for Cell and Gene Therapy, Leiden University Medical Center, Albinusdreef 2, 2333 ZA, Leiden, the Netherlands

**Keywords:** Controlled human infection model, Neglected tropical diseases, Schistosomiasis, *Schistosoma mansoni*

## Abstract

**Background:**

A controlled human infection model for schistosomiasis (CHI-S) can speed up vaccine development and provides insight into early immune responses following schistosome exposure. Recently, we established CHI-S model using single-sex male-only *Schistosoma mansoni (Sm)* cercariae in *Schistosoma*-naïve individuals. Given important differences in antigenic profile and human immune responses to schistosomes of different sex, we pioneered a single-sex female-only CHI-S model for future use in vaccine development.

**Methods:**

We exposed 13 healthy, S*chistosoma*-naïve adult participants to 10 (n = 3) or 20 (n = 10) female cercariae and followed for 20 weeks, receiving treatment with praziquantel (PZQ) 60 mg/kg at week 8 and 12 after exposure.

**Findings:**

The majority (11/13) participants reported rash and/or itch at the site of exposure, 5/13 had transient symptoms of acute schistosomiasis. Exposure to 20 cercariae led to detectable infection, defined as serum circulating anodic antigen levels >1.0 pg/mL, in 6/10 participants. Despite two rounds of PZQ treatment, 4/13 participants showed signs of persistent infection. Additional one- or three-day PZQ treatment (1 × 60 mg/kg and 3 × 60 mg/kg) or artemether did not result in cure, but over time three participants self-cured. Antibody, cellular, and cytokine responses peaked at week 4 post infection, with a mixed Th1, Th2, and regulatory profile. Cellular responses were (most) discriminative for symptoms.

**Interpretation:**

Female-only infections exhibit similar clinical and immunological profiles as male-only infections but are more resistant to PZQ treatment. This limits future use of this model and may have important implications for disease control programs.

**Funding:**

European Union's 10.13039/501100007601Horizon 2020 (grant no. 81564).

**Research in context**

**Evidence before this study**

A controlled human infection model for schistosomiasis has the potential to speed up vaccine development and provide insight into early immune responses following schistosome exposure. Previously, a male-only *Schistosoma mansoni (Sm)* infection model has been successfully developed in *Schistosoma*-naïve participants, in which exposure to male cercariae led to detectable infection and in which all were successfully cured after 1–2 doses of praziquantel. However, animal studies suggest there are significant differences between immune responses to male and female worms, and differential expression of some vaccine antigens in male and female adult worms suggests important differences in the use of single-sex models for vaccine testing.

**Added value of this study**

This study aimed to establish a controlled human *Schistosoma mansoni* infection with female cercariae. We show that exposure to 20 female cercariae leads to detectable infection and is well-tolerated, however resulting infections are refractory to (repeated) praziquantel treatment. We observe strong similarities between immune responses in male- and female-only infections, which show a mixed Th1/Th2 immunophenotype contrasting the existing dogma that Th2 responses result from egg deposition.

**Implications of all the available evidence**

Female-only infections exhibit similar clinical and immunological phenotypes profiles, but are more resistant to PZQ treatment than male-only infections. This limits future use of this model for vaccine testing, but may have important implications for disease control programs.

## Introduction

Schistosomiasis, an infection with *Schistosoma* parasites, remains an important neglected tropical disease (NTD) that adversely affects global health with an estimated prevalence of 240 million.^1^ Infection occurs through contact with fresh water that contains cercariae, the larval stage of *Schistosoma,* which are secreted by freshwater snails. Subsequent egg deposition by the matured male and female worm pair is responsible for schistosome morbidity as it causes local inflammation and granulomas.^2^ Control of disease relies heavily on mass drug administration (MDA) with praziquantel (PZQ), however reinfections occur rapidly. Modelling studies suggest that an efficacious vaccine against schistosomiasis is needed to improve disease control.^3^ Currently, four vaccine candidates are in clinical testing.^4^ A controlled human infection with *Schistosoma* (CHI-S), whereby healthy adult participants are deliberately exposed to schistosomes, may be instrumental to get an early vaccine efficacy estimate and guide vaccine selection and design for larger phase 3 studies.^5^ To avoid egg-induced pathology, we previously developed a single-sex, CHI-S model with male-only, *Schistosoma mansoni (Sm)* cercariae. In this study, exposure of *Schistosoma*-naïve participants to 20 male cercariae led to detectable infection by worm-derived circulating anodic antigen (CAA) in 9 out of 11 participants (82%) before clearing infection with one to two doses of PZQ.^6^ However, animal studies suggest that there are significant differences between immune responses to male and female worms.^7^ In addition, differential expression of vaccine antigens in male and female adult worms may require the development of CHI-S for both schistosome sex for testing of vaccines targeting adult worm antigens.^8^ We thus aimed to develop a single-sex, female CHI-S model complementary to the previous male model to enable exploration of sex-specific immune responses and future vaccine testing.

## Methods

### Study design

This open label, dose-escalation study (clinicaltrials.gov identifier: NCT04269915) took place at the Leiden University Medical Centre, The Netherlands between September 2020 and April 2022. Healthy, 18–45 year old *Schistosoma*-naïve participants without prior (suspected) exposure to schistosomes and without travel plans to *Schistosoma*-endemic regions during the study period were recruited through advertisements. Participants were excluded if they had a history or evidence of any illness that could compromise the health of the participant during the study or affect the interpretation of study results. Participants with a known hypersensitivity to or contra-indications for use of PZQ, artesunate or lumefantrine were also excluded. Participant's sex was self-reported.

### Ethics

The study was approved by the local medical ethics review board (METC-LDD: P20.015) and was conducted in accordance with ICH-GCP guidelines and the Declaration of Helsinki. All participants provided written informed consent for participation.

### Study procedures

After inclusion, small groups of three people were then exposed to a pre-defined number of female *Sm* cercariae. The doses were based on our previous study with male *Sm* cercariae. Depending on safety and infectivity, dose was either escalated or an additional seven participants were exposed to the same dose after discussions with the safety monitoring committee (SMC), taking into account safety data from the study with male cercariae. Sample size was based on the number of other proof-of-concept vaccine efficacy studies, where small groups of 10 subjects are preferred.

Female *Sm* cercariae were produced as described previously,^9^ with the addition of a confirmatory PCR on 10 individual, hand-picked cercariae from each snail to ascertain female-only cercariae. Cercariae were applied in 0.5 mL mineral water to the skin of the participant's forearm for 30 min. Next, microscopy was performed on rinse water to count the remaining cercarial tails.

Participants were followed up weekly until week 16 for adverse event and sample collection. PZQ treatment (60 mg/kg) was provided at week 8 and 12. Long-term visits were at week 18, week 20 and week 52 after infection.

### Outcomes

Safety was assessed through adverse events (AEs) reporting and blood tests. Severity of adverse events was assessed as follows: symptoms that do not interfere with daily activities (mild); symptoms that interfere with or limits daily activities (moderate); and symptoms resulting in absence or required bed rest (severe). AEs were assessed as related or unrelated to study procedures based on clinical judgement. Symptoms of acute schistosomiasis were defined as: fever, urticaria, angioedema, night sweats, myalgia, arthralgia, dry cough, diarrhoea, abdominal pain, and headache occurring between 2 and 8 weeks after exposure without other clear cause. Classification of acute schistosomiasis (yes/no) was performed separately by two clinicians and in case of disagreement, consensus was reached after discussion.

Patent infection was determined by upconverting reporter particle lateral flow (UCP-LF CAA) assay in serum^10^ and defined by at least one value ≥ 1.0 pg/mL before week 8. Values below the limit of detection (<0.5 pg/mL) were set to 0.25 pg/mL. In addition, schistosome-specific antibodies were measured using in-house adult worm IgM antibody (IFA) and soluble egg IgG antibody (ELISA) assays.^11^ To rule out egg production, *Schistosoma* PCR was performed on faeces.^12^ Adult worm antigen (AWA)-specific IgG, IgG1, and IgE were measured by ELISA as previously described^6^ with the following modifications: 1) plates were coated with 25 μg antigen and 2) pooled positive participants from the previous CHI-S was used as standard.^6^

Serum samples were assessed for CCL2, CXCL10, IL-4, CCL4, IL-1β and IL-10 using a custom Luminex kit (LXSAHM-06, R&D systems) according to manufacturer instructions. Cytokines with over 40% of samples under the limit of detection were not included in the analysis (IL-4, IL-1β and IL-10).

PBMCs were isolated using a ficoll gradient, cryopreserved, thawed and stimulated with AWA, media or SEB for 24 h as previously described [6]. Cells were centrifuged at 400 g for 4 min, supernatants were cryopreserved at −80 °C for cytokine analysis using a Luminex kit (LKTM008, R&D systems) per manufacturer's instructions. The following cytokines were measured: GM-CSF, IFNγ, IL-1β, IL-2, IL-4, IL-5, IL-6, IL-10, IL-12-p40, IL-13 and TNFα. IL-12p40 was excluded from further analysis as all samples were below the LOD. Antibody staining and flow cytometry of cells was performed as previously described [6]. Antibodies used in flow cytometry staining were FcBlock (BD biosciences, 1:100, 564,219), CD3 (APC-ef780, eBioscience, 1:800, 47-0038-42), CD4 (PE-Cy5, eBioscience, 1:400, 555,348), IFNγ (BV421, BioLegend, 1:1000, 502,531), IL-2 (FITC, FITCXBD biosciences, 1:25, 340,448), Th2-cytokines IL-4 (PE, BD biosciences, 1:20, 340,451), IL-5 (PE, BioLegend, 1:250, 504,303), IL-13 (PE, BioLegend, 1:100, 501,903), TNFα (PE-Cy7, eBioscience, 1:1000, 25-7349-41), IL-10 (BV711, BD biosciences, 1:250, 564,050), Foxp3 (PE-CF594, BD biosciences, 1:100, 562,421) and CD25 (BD biosciences, 1:800, 340,907). All antibodies are available commercially and validated by the referenced supplier. Data was analysed with FlowJo 10.8, gating scheme in Supplementary Fig. S1. FMO controls were used for gating, with SEB as a positive control.

### Statistics

All 13 participants were included in the intention-to-treat analysis. Given the low number of participants, adverse event, CAA, and antibody data were mostly descriptive and no formal statistical testing was used to compare dose groups. Data analyses and visualisation was performed using R (v4.2) and R studio (v2022.02.3). A Kaplan Meier plot was used to describe when participants first become CAA positive. For cytokine analysis, linear mixed models were fitted to compare mean responses within participants over time, with participant as a random effect and time in weeks as a fixed effect (as a factor; weeks 0,4,8,12) using the packages lme4 (version 1.1–29) and lmerTest (version 3.1–3). No other variables, such as dose group, were included in the model. Model assumptions of normality and homogeneity of residuals were checked through residual plots.

Data integration was performed using the ‘mixOmics’ package in R (version 6.8.0).^13^ For comparability, data from both male^6^ and female models were separately centred and scaled using the base R scale function. Multiblock sparse partial least squares discriminant analysis (multiblock sPLS-DA) was used to identify correlated variables in multiple dataset ‘blocks’ predictive of acute schistosomiasis symptoms.^13^ Feature selection was performed using Lasso-like penalization for all analyses. For both the male-only and combined model the number of components was set to two and tuned to determine the features per block (1–4 per component). Correlations between blocks of 0–1 were trialled, with 0.75 and 1 chosen because of the lowest error rate for the combined and male-only models. For the combined model 3,4,6 and 5 features were chosen from the Luminex, Antigens, Antibodies and Cellular blocks respectively. For the male-only model 4,4,5 and 2 features were chosen from the Luminex, Antigens, Antibodies and Cellular blocks respectively. Weighted votes consider correlation between the latent components of the block and the predicted outcome.

### Role of funders

The funder of the study (European Union) had no role in study design, data collection, data analysis, data interpretation, writing the manuscript, and the decision to submit.

## Results

**Fig. 1:**
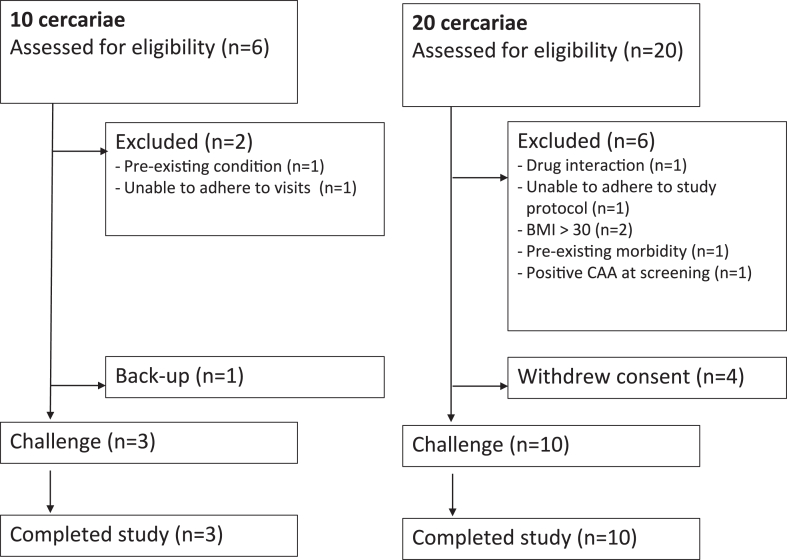
**Consort flow diagram of study participants**. Participants were exposed to either 10 (n = 3) or 20 (n = 10) female cercariae.

Out of 26 individuals screened for eligibility, 13 were included. In line with the adaptive dose design, three were exposed to 10 female *Sm* cercariae, while the remaining ten were exposed to 20 female *Sm* cercariae (Fig. 1) after discussions with the SMC. There was no loss to follow-up. Eight (62%) participants were female (Table 1). Median age at infection was 26 years (range: 18–38). Immediately after exposure, rinse water was examined and showed very few remaining cercarial heads (median 0, range 0–2) or whole cercariae (median 0, range 0–1), but many cercarial tails (median 7, range 4–13), suggesting successful skin penetration (Supplementary Table S1).

**Table 1:** Baseline characteristics of study participants.

	All (n = 13)	10 Cercariae group (n = 3)	20 Cercariae group (n = 10)
Age in years, median (range)	26 (18–38)	33 (22–38)	26 (18–35)
Sex, n (%)			
Male	5 (38%)	1 (33%)	4 (40%)
Female	8 (62%)	2 (67%)	6 (60%)

**Fig. 2:**
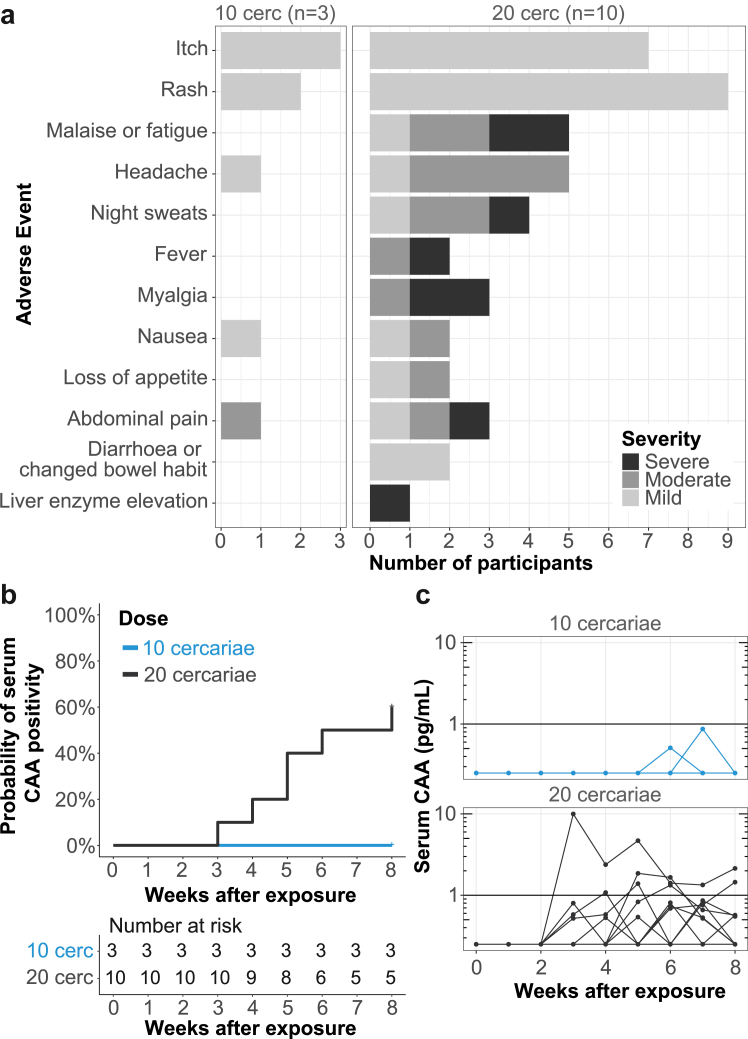
**Safety and infectivity of exposure to female cercariae**. The number of participants developing *Schistosoma*-related symptoms (possibly, probably or definitely related) in the eight weeks following exposure by dose and severity **(a)**. Reverse Kaplan–Meier plot for the probability of CAA positivity (CAA ≥1.0 pg/mL, at any time point after exposure) over time per dose group **(b)**. Individual trajectories for CAA levels **(c).** CAA values < 0.5 pg/mL (LoD) were set to 0.25 pg/mL. Horizontal line at 1.0 pg/mL shows the CAA cut-off for CAA positivity. Cercs = cercariae.

No serious adverse events were reported. Adverse events related to *Schistosoma* infection occurred in all participants (Fig. 2) of which more than half (55%) were mild. Most participants developed a local skin reaction at the site of infection and reported rash (11/13, 85%) and itching (10/13, 77%) shortly after exposure. While itching resolved within 1–2 days, rash remained visible for median 23 days (range: 1–42). From week three onwards, systemic adverse events occurred (Supplementary Fig. S2) in 5/10 participants exposed to 20 cercariae, indicative of acute schistosomiasis. One participant experienced moderate symptoms and four participants experienced severe symptoms (median duration: 2 days) that could effectively be managed with common analgesics (paracetamol and NSAIDs). One participant had prolonged symptoms that required treatment with prednisolone, after which symptoms swiftly resolved. During this period, this participant also showed transient elevations of liver enzyme tests (ALT >5 x ULN) without focal abnormalities on abdominal ultrasound, which over the course of three weeks also resolved. Apart from common PZQ side effects, no related adverse events were reported after week 8. Eosinophil counts for both dose groups peaked between 5 and 8 weeks after exposure (Supplementary Fig. S3).

None of the participants exposed to 10 female cercariae showed patent infection (CAA values ≥ 1.0 pg/mL) at any time point after exposure, whereas in those exposed to 20 cercariae six (out of 10, 60%) did starting at three weeks after exposure (Fig. 2). *Schistosoma* PCR on faeces at week 8 was negative for all participants indicating no eggs were produced.

**Fig. 3:**
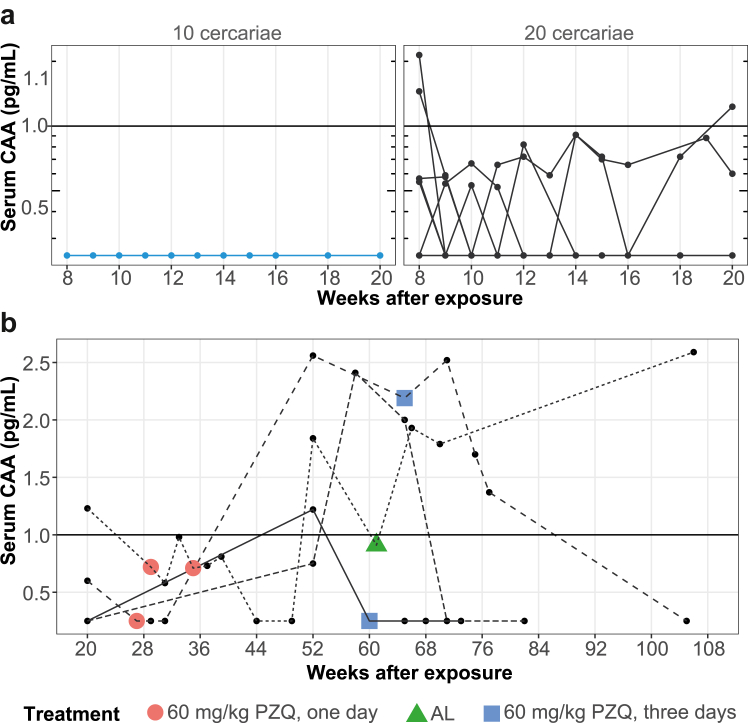
**Serum CAA levels after treatment with PZQ**. Individual CAA trajectories after treatment with PZQ 60 mg/kg at week 8 and 12 for each dose group **(a)**. Overview of additional treatment regimens in 4 participants with persisting CAA levels **(b)**. CAA values < 0.5 were set to 0.25. Horizontal line at 1.0 pg/mL shows the cut-off for CAA positivity. PZQ: praziquantel. AL: artemether-lumefantrine.

After PZQ 60 mg/kg treatment at week 8 and 12, four (out of 10) participants in the 20 cercariae group showed signs of persistent infection without any symptoms (Fig. 3) at the one-year follow-up timepoint. Three were CAA positive (≥1.0 pg/mL) infection, while one had an indeterminate result (CAA between 0.5–1.0 pg/mL). Remarkably, serum CAA levels first fell to below detection limit to then recur. Because repeated treatment with PZQ as a single day schedule was unsuccessful in these participants, a three-day schedule with 60 mg/kg PZQ split into three doses (morning, afternoon, and evening, 20 mg/kg each) was tried in two participants. One of these participants had self-cured already at the time of PZQ distribution and the other was not cured after the three-day course. Another participant was treated with artemether (artemether/lumefantrine 20/120 mg, 24 tablets split in 6 equal doses at time = 0, 8, 24, 36, 48, and 60 h), as artemisinin derivatives have reported to be active also against immature worms.^2^ Artemether/lumefantrine is the only oral artemisinin derivative registered in The Netherlands. Unfortunately, this also did not result in cure. After one-year additional follow-up three participants had self-cured, while the remaining participant remains under follow-up.

**Fig. 4:**
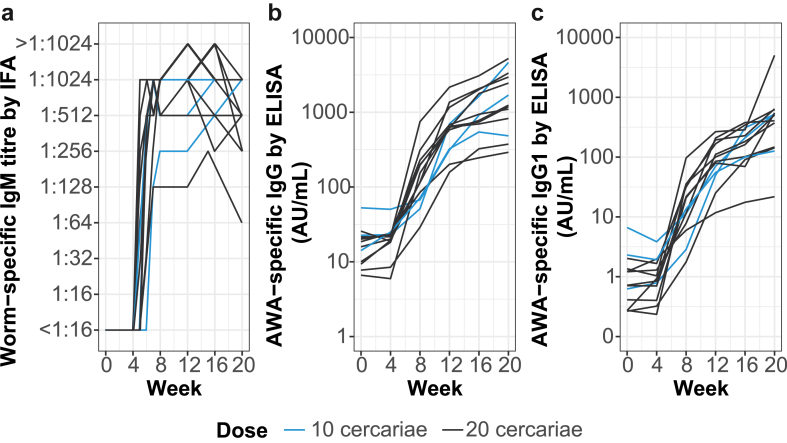
**Antibody responses after exposure to female cercariae**. Plots show individual changes in antibody levels over time in worm-specific IgM **(a)**, AWA-specific IgG **(b)**, and AWA-specific IgG1 **(c)**.

All participants seroconverted for adult-worm IgM between 4 and 7 weeks after exposure (Fig. 4a), using IFA. Also, total IgG and IgG1 against AWA measured by ELISA increased from week 8 onwards (Fig. 4b and c), while no changes in IgG4 and IgE against AWA were observed (Supplementary Fig. S4). Two out of 13 and 10 out of 13 participants showed detectable IgG against soluble egg antigen (SEA) at week 20 and 52, respectively.

Cytokines and chemokines in serum revealed an increase in the pro-inflammatory chemokines CXCL10 and CCL4 at week 4 post infection (Supplementary Fig. S5a). Other serum cytokines measured were either unchanged during infection (CCL2) or below the limit of detection (IL-4, IL-1β and IL-10).

To understand the development of cellular *S. mansoni* specific immune responses peripheral blood mononuclear cells (PBMCs) were stimulated with AWA and both intracellular (Supplementary Fig. S5b) and secreted (Supplementary Fig. S5c) cytokines assessed. At week 4 post infection, CD4^+^ T-cells showed a mixed response to *S. mansoni* AWA by secreting cytokines which encompassed Th1 (IFNγ, TNFα, IL-2 & IL-1β), Th2 (IL-4, IL-5, IL-13) and regulatory responses (IL-10) as well as the regulatory T cell transcription factor Foxp3. AWA-induced Th2 cytokine and Foxp3 expression remained significantly elevated to week 8 post infection.

**Fig. 5:**
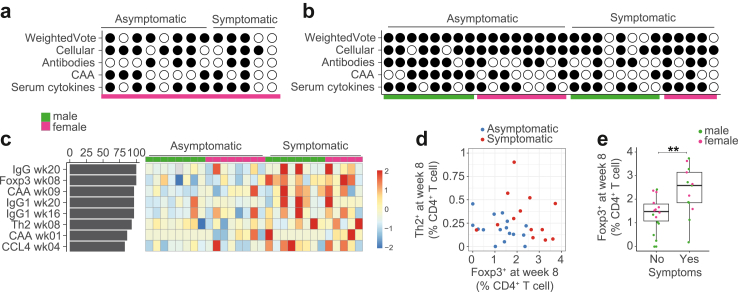
**Data integration and symptom prediction. (a–b)** Filled circles indicate a correct prediction and open circles a false prediction of acute schistosomiasis using multiblock sPLS-DA. Individual participants shown in columns, blocks in rows. **(a)** Capacity of multiblock sPLS-DA trained on male-only infection data to predict symptoms during female-only infection (n = 13). **(b)** Capacity of multiblock sPLS-DA trained on combined male and female infection (n = 29) to predict symptoms, result of leave one out cross validation (LOO-CV). **(c)** Consensus features present in over 75% of LOO-CV models displayed. The z score-normalized levels of chosen features are indicated in the heat map, one column corresponds to one participant. **(d)** Scatterplot of AWA-specific Foxp3 and Th2 cytokine expression at week 8. Points represent individual participants, coloured by symptoms. **(e)** Boxplot displays Foxp3 levels at week 8 in the combined studies, split by symptoms. For plotting, negative values have been placed at zero. Points represent individual participants, coloured by study, showing median, interquartile range, with whiskers extending to the largest or smallest value no further than 1.5X the interquartile range.

Next, we wanted to understand how host and parasite parameters were associated with the occurrence of acute schistosomiasis syndrome. In line with our previous study, we used multiblock sparse partial least squares discriminant analysis (multiblock sPLS-DA), to identify correlated variables in multiple dataset ‘blocks’ predictive of acute schistosomiasis symptoms.^13^ Using immunological and parasitological parameters from male-only infection as a training set, we were able to correctly discriminate acute schistosomiasis in 9/13 (69%) of female-only infected participants (Fig. 5a). To improve performance of this model and potential generalizability to future studies we combined both male and female only infection studies into one multiblock sPLS-DA (Fig. 5b). This combined model assigned 25 of 29 participants (86%) correctly (Fig. 5b). Serum CAA and AWA-specific antibodies were poorly discriminative for symptoms. In contrast, serum cytokines performed better (65%) and the cellular block performed as well as the combined model (86% correct) (Fig. 5b). Important variables, chosen in over 75% of the LOO models, are shown in Fig. 5c. Our findings were confirmed by separate sPLS-DA analysis per block, the cellular block remaining the most discriminative (83% correct) (Supplementary Fig. S6). AWA-specific Th2 cytokines and Foxp3 expression at week 8, the two features chosen in the highly discriminative cellular block of the male and female model (Fig. 5b and c), together could separate the majority of symptomatic from non-symptomatic individuals (Fig. 5d). In line with this, there were significantly higher Tregs at week 8 in individuals with acute schistosomiasis (Fig. 5e).

## Discussion

In this study, we established a single-sex female, *Schistosoma mansoni* controlled human infection model with 60% of participants showing serum CAA detectable infection after exposure to 20 female cercariae. Transient local (rash and itch) and systemic (acute schistosomiasis) symptoms were observed. The resulting female-only infections were refractory to treatment with PZQ. Despite double treatment with PZQ, four (out of 13) participants showed signs of persistent infection, all after exposure to 20 cercariae. Additional one- or three-day PZQ treatment (1 × 60 mg/kg and 3 × 60 mg/kg) or artemether did not result in cure, but over time three participants self-cured.

Based on animal studies, unpaired female worms are thought to remain immature and therefore excrete lower amounts of gut-derived antigens.^14^ However, we did not find evidence of reduced CAA secretion or symptoms in this small group of individuals. Similar attack rates were observed for exposure to 20 female cercariae (60%, 6 out of 10) and 20 male cercariae (82%, 9 out of 11).^6^ Moreover, exposure to female cercariae resulted in similar symptoms as in the single-sex male infection study, with nearly all participants having local skin reactions and roughly 50% developing symptoms of acute schistosomiasis syndrome in the 20 cercariae group, similar to 55% after exposure to 20 male cercariae.^6^ These symptoms could effectively be managed with standard analgesics and prednisolone. Although this risk of challenge-related symptoms was substantial, symptoms are well-tolerated and of short duration without irreversible harm in line with other controlled human infection model studies.^15^

Immune responses to female-only infection were markedly similar to our previous findings during male-only schistosome infection. As observed in male-only infection, antibody responses to female-only infection were characterised by increases in worm-specific IgM, AWA-specific total IgG and IgG1 after four weeks, but not IgE. Cellular and cytokine responses peaked at week 4 post infection, with a mixed response encompassing Th1, Th2 and regulatory profiles.^6^ These findings contrast the dogma that schistosome infection induces initial Th1 responses, with Th2 responses induced upon egg deposition,^16^ instead supporting more recent evidence of mixed Th1/Th2 responses to maturing schistosomes.^17^ The similarity between male- and female-induced immune responses is in line with recent work using long-term male and female schistosome infections in a murine model.^18^ However, it contrasts prior literature which suggested male-only infections induce a more pro-inflammatory response, whilst female-only infection are characterized by more regulatory profiles.^7^^,^^19^ However, given the very small set of individuals in both our male- and female-only CHI-S studies, more in-depth immunological assessments will be needed to confirm similarity of immune interaction between male and female worms.

We did not find evidence for (unfertilised) egg excretion in stool by PCR, but did observe egg-specific IgG at later time points (week 20 and 52) which may be suggestive of antibody cross-reactivity, similar to the male-only study, to for instance glycan epitopes that are shared between cercariae and eggs.^20^ However, we cannot entirely rule out the presence of unfertilised eggs below the PCR detection threshold or that do not penetrate the gut. In contrast to male-only infections where egg production has not been reported, we and others have in rare occasions observed egg production in single-sex female infection of mice.^14^^,^^21^ The number of eggs recovered in mice were negligible compared to mixed-sex infections and were never found to contain a viable miracidium.

Integrating immune and parasitological parameters with acute schistosomiasis symptoms revealed that symptoms were related to interindividual variability in host responses rather than the levels of active infection as measured by CAA. Long-lasting (week 8) AWA-specific Treg expansion was particularly discriminatory for symptomatic individuals. We suggest that both week 8 Treg expansion, and week 4 symptoms may result from enhanced inflammation at week 4 in symptomatic individuals, supported by a trend for increased inflammatory chemokine (CCL4) in symptomatic individuals, however further immunological characterisation is required to establish a definitive causal relationship. Tregs have been shown to be elevated during endemic *S. mansoni* infection,^22^ with murine models revealing a crucial role for Tregs in reducing immune pathology following egg-production.^23^^,^^24^ Whether the enhanced AWA-specific Treg response found in symptomatic individuals in CHI-S could dampen immune responses to subsequent schistosome infection remains to be investigated.

Even though individuals were treated twice with PZQ, infection could not be fully cleared in four out of six individuals. Moreover, repeated treatment with a one- and or three-day PZQ or artemether-lumefantrine did not lead to cure, however three participants self-cleared within 2 years after exposure. The remaining participant, although showing low CAA levels, just above the limit of detection, will remain under follow-up and is also expected to self-clear. Unfortunately, oxamniquine which is also used to treat *Sm* infecton is not registered nor available in The Netherlands and could therefore not be used. Decreased drug susceptibility of single-sex females to PZQ had previously only been reported in animal models and is not well understood.^25^^,^^26^ One of the explanations proposed is the aforementioned incomplete maturation of the female in the absence of a male worm which would result in decreased sensitivity. Artemisinin derivatives have reported efficacy against immature stages of the worm,^27^ but in our participant did not lead to cure. Another explanation for the reduced susceptibility may be the location of the worms: single-sex female worms may be unable to migrate to the mesenteric vessels and stay in the liver, where the effective drug concentration is lower due to high first-pass effect of PZQ. Other individual pharmacogenetic factors, particularly in drug-metabolising cytochrome P450 enzymes, have also been found to influence effective drug concentrations,^28^ however given the high frequency of PZQ failure in our study, this seems unlikely as a single cause of drug failure. Despite the excellent safety profile, the lack of cure after PZQ treatment unfortunately precludes the use of the female-only CHI-S model at a larger scale.

The decreased susceptibility of unpaired female worms to PZQ may also have important implications for schistosomiasis control programs in endemic areas. Even though the frequency of single-sex infection in nature is unclear, it seems likely unpaired female worms can survive treatment and can persist for over two years. Recently, Winkelmann et al. hypothesised that female worms may have developed more effective evasion strategies than males as demonstrated through repulsion of the opsonized surface, faster regeneration after PZQ, and upregulation of gene expression associated with tegument maintenance after incubation with serum.^29^ This was observed in paired (and separated) female worms from a mixed-sex infection as well as in unpaired female worms from a single-sex infection. If natural resistance of female worms to PZQ indeed also holds true for paired worms, surviving females may explain the findings of low cure rates, such as a recent study in schoolchildren investigating repeated PZQ treatment, which found a considerable decrease in CAA in urine but a lack of clearance.^30^

An important limitation of this study involves the small sample size and the intrinsic difference to natural, mixed-sex egg-producing infections. Nevertheless, we were able to gain valuable insights into host responses following exposure to female cercariae, which were clinically and immunologically very similar to those in the male-only model. Moreover, this study shows decreased susceptibility of female worms to PZQ in the human host which limits the use of this model for future vaccine testing but may have important implications for disease control programs.

## Contributors

MR acquired funding. JK, MR prepared the research protocol. JK, MR, CH, MY. were involved in study design. JS, MC, IvA, PM, AvD, HH were involved in production and release of cercariae. JK, EH, JJ, MC, OL, JS, CD, SH, AO, VK, GR, LdB, YK generated the data. LvL, LW, GvD, PC were involved in the infection endpoint measurements and interpretation. JK, JJ were involved in data curation, project administration, and accessed and verified the data. JK, EH performed the data analyses and prepared the first draft. SJ provided support for mixomics analyses. All authors have read and approved the final version of the manuscript. All authors have confirmed full access to all data in the study and were responsible for the decision to submit the manuscript for publication.

## Data sharing statement

After publication, all data will undergo FAIRification and will be made available anonymised through a LUMC-based fair data point which will be made accessible through data visiting. The study protocol is available as supplementary material with this publication.

## Declaration of interests

Authors declare that they have no competing interests.

## Appendix A Supplementary data

Supplementary Table and Figs

Protocol
