# Supplementary material for: Safety and infectivity of female cercariae in Schistosoma-naïve, healthy participants: a controlled human Schistosoma mansoni infection study
Source: eBioMedicine. 2023 Oct 12;97:104832. doi: 10.1016/j.ebiom.2023.104832 (PMC10585222; doi:10.1016/j.ebiom.2023.104832)
Supplement: Supplementary Table and Figs [file mmc1.pdf]

Supplementary material

Contents

Supplementary Fig. 1. Flow cytometry gating strategy 2

Supplementary Fig. 2. Timing of related adverse events after challenge for all participants. 3

Supplementary Fig. 3. Eosinophil counts after exposure to female cercariae. 4

Supplementary Fig. 4. Antibody responses after exposure to female cercariae. 5

Supplementary Fig. 5. Serum cytokines, AWA induced intracellular and secreted cytokines. 6

Supplementary Fig 6. Individual sPLS-DA prediction analyses 7

Supplementary Table 1. Microscopy counts of cercariae in rinse water after exposure 8

Supplementary Fig. 1. Flow cytometry gating strategy

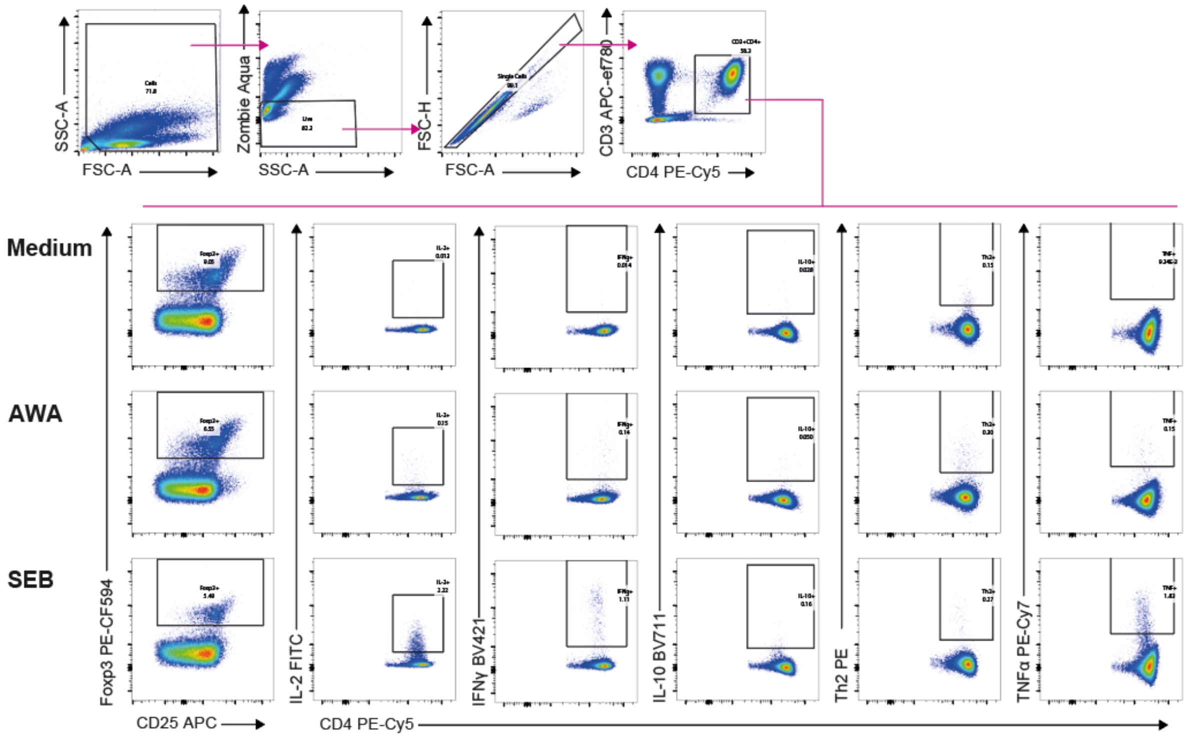

Initial gates chose cells, live cells, single cells and then CD4$^+$ CD3$^+$ T cells. CD4$^+$ CD3$^+$ T cells were then gated on Foxp3, IL2, IFNγ, IL10, Th2 (IL-4, IL-5, IL-13) or TNFα, positive cells. Initial gates are shown from medium stimulated cells. Foxp3 and cytokine gates show cells stimulated with either medium, Staphylococcal Enterotoxin B (SEB) or adult worm antigen (AWA). Magenta arrows relate to order of gating, numbers in plots are frequencies of parent population.

Supplementary Fig. 2. Timing of related adverse events after challenge for all participants.

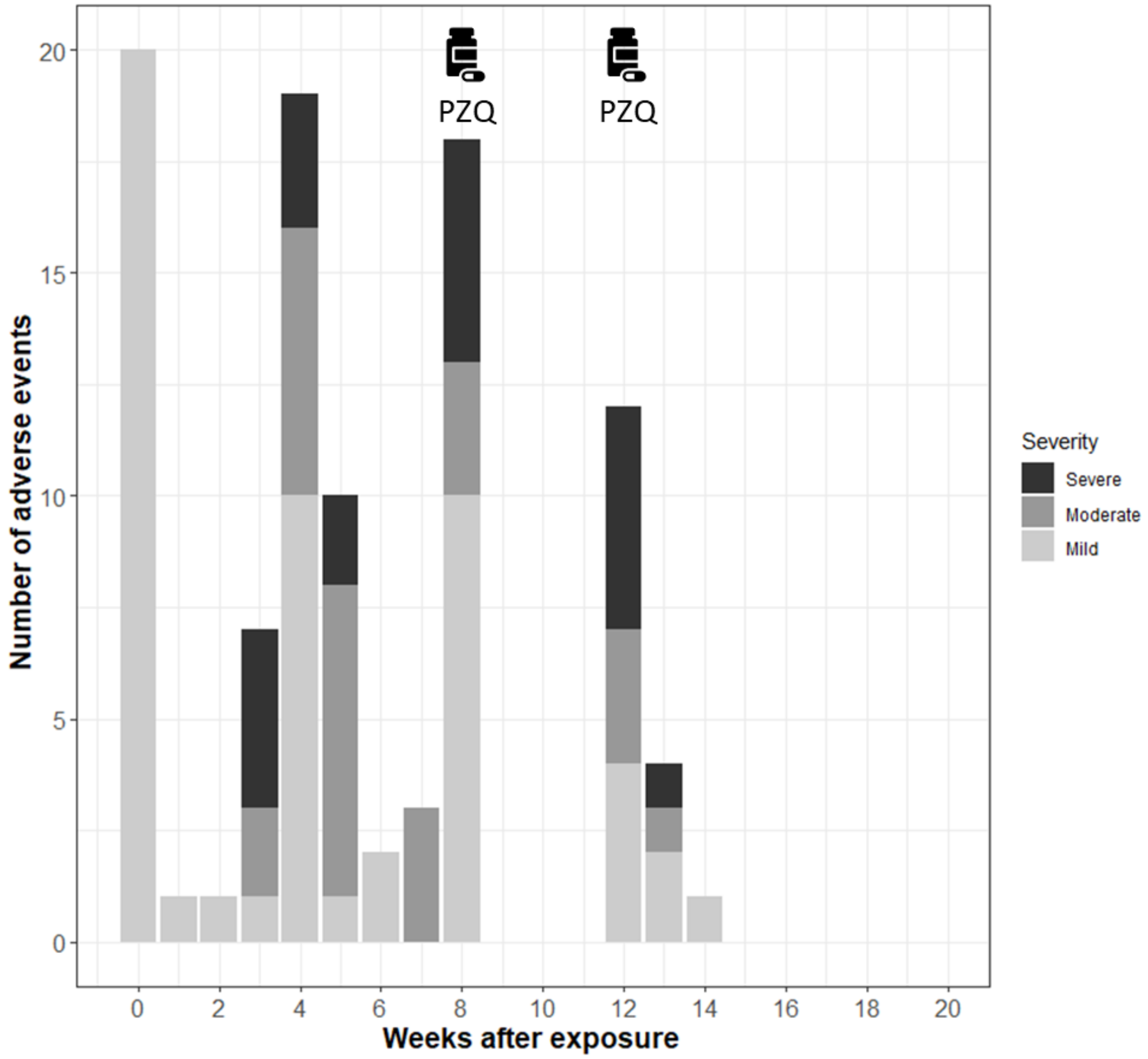

This figure shows that a large number of AEs are reported at week 0 (itch and rash). Another increase in reported AEs occurs between weeks 3-5 which correspond to the onset of acute schistosomiasis. Note these only occurred in those exposed to 20 cercariae. High number of adverse events reported at week 8 and 12-14 are common side effects of praziquantel treatment. PZQ: praziquantel

Supplementary Fig. 3. Eosinophil counts after exposure to female cercariae.

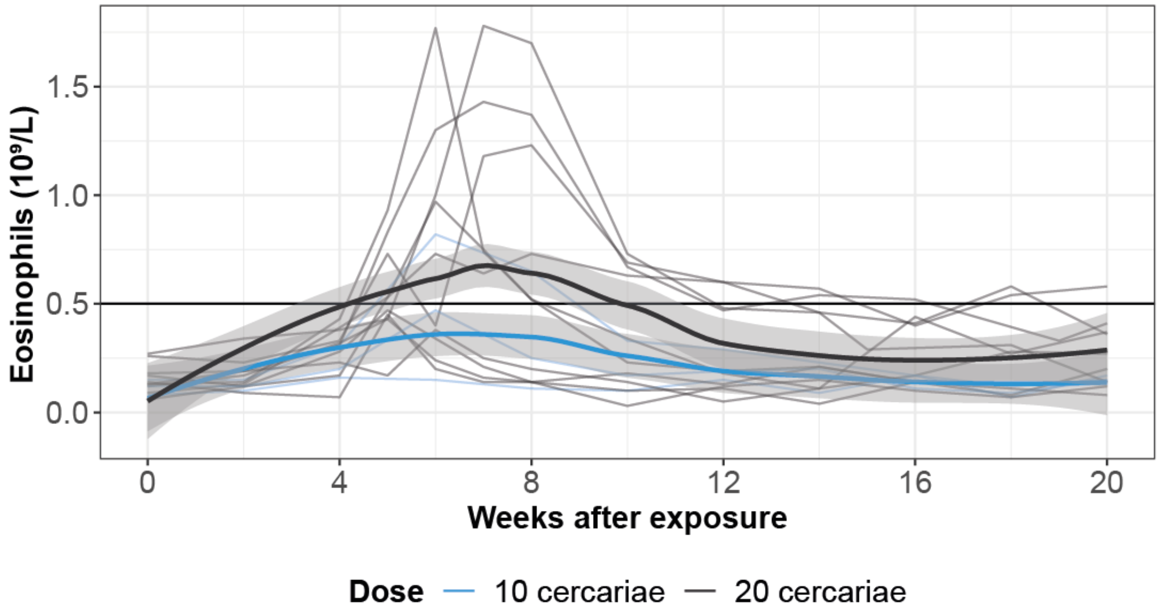

Graph shows the kinetics in eosinophil counts for the two dose groups. The light blue and light grey lines show the individual trajectories, whereas the bold lines show the locally weighted smoothing (LOESS) line with 95% confidence interval. Horizontal line at 0·5 * 10^9 eosinophils/L shows the clinical cut-off for abnormality.

Supplementary Fig. 4. Antibody responses after exposure to female cercariae.

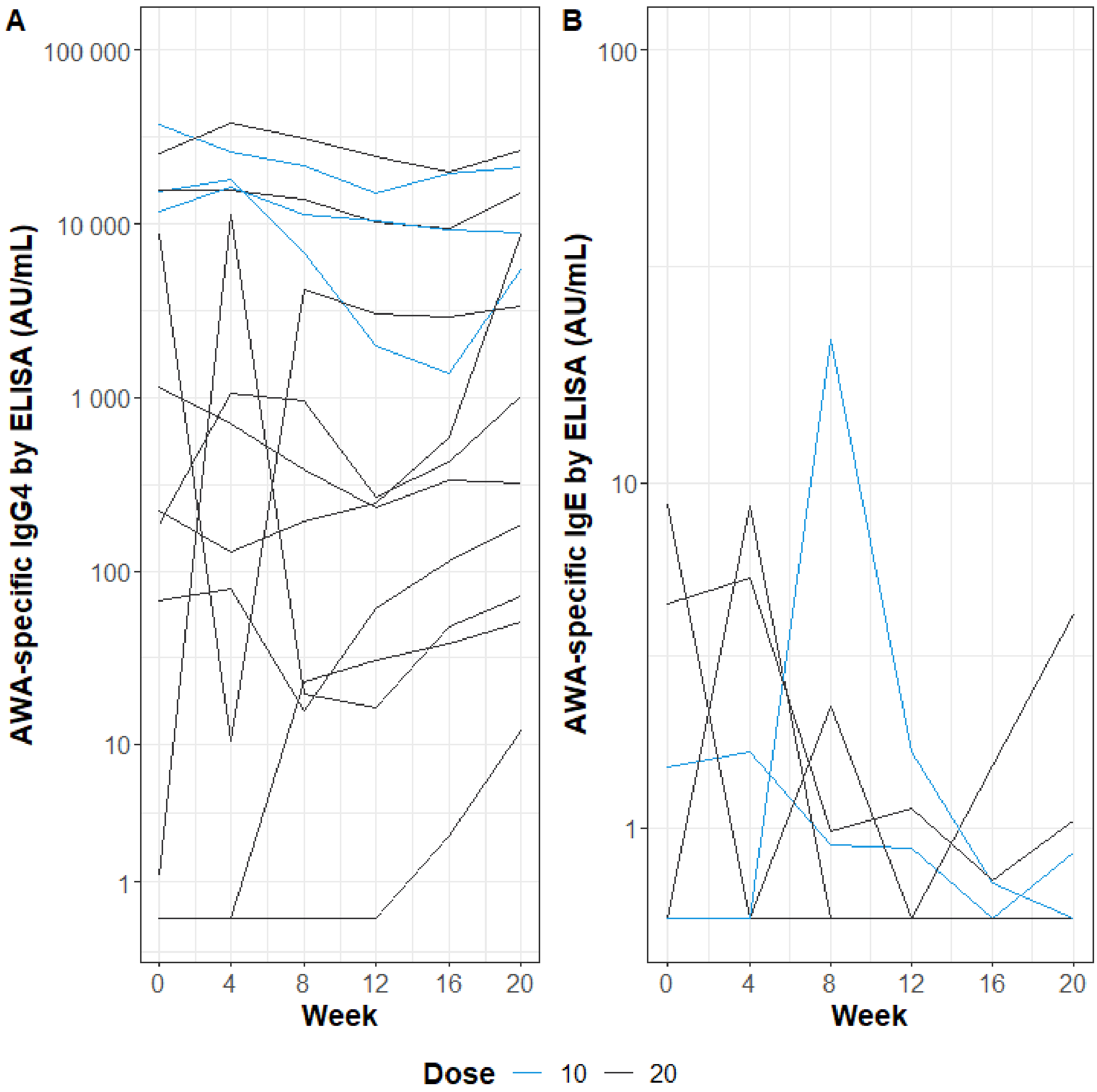

Plots show individual changes in antibody levels over time in worm-specific IgG4 **(A)**, and AWA-specific IgE **(B).**

Supplementary Fig. 5. Serum cytokines, AWA induced intracellular and secreted cytokines.

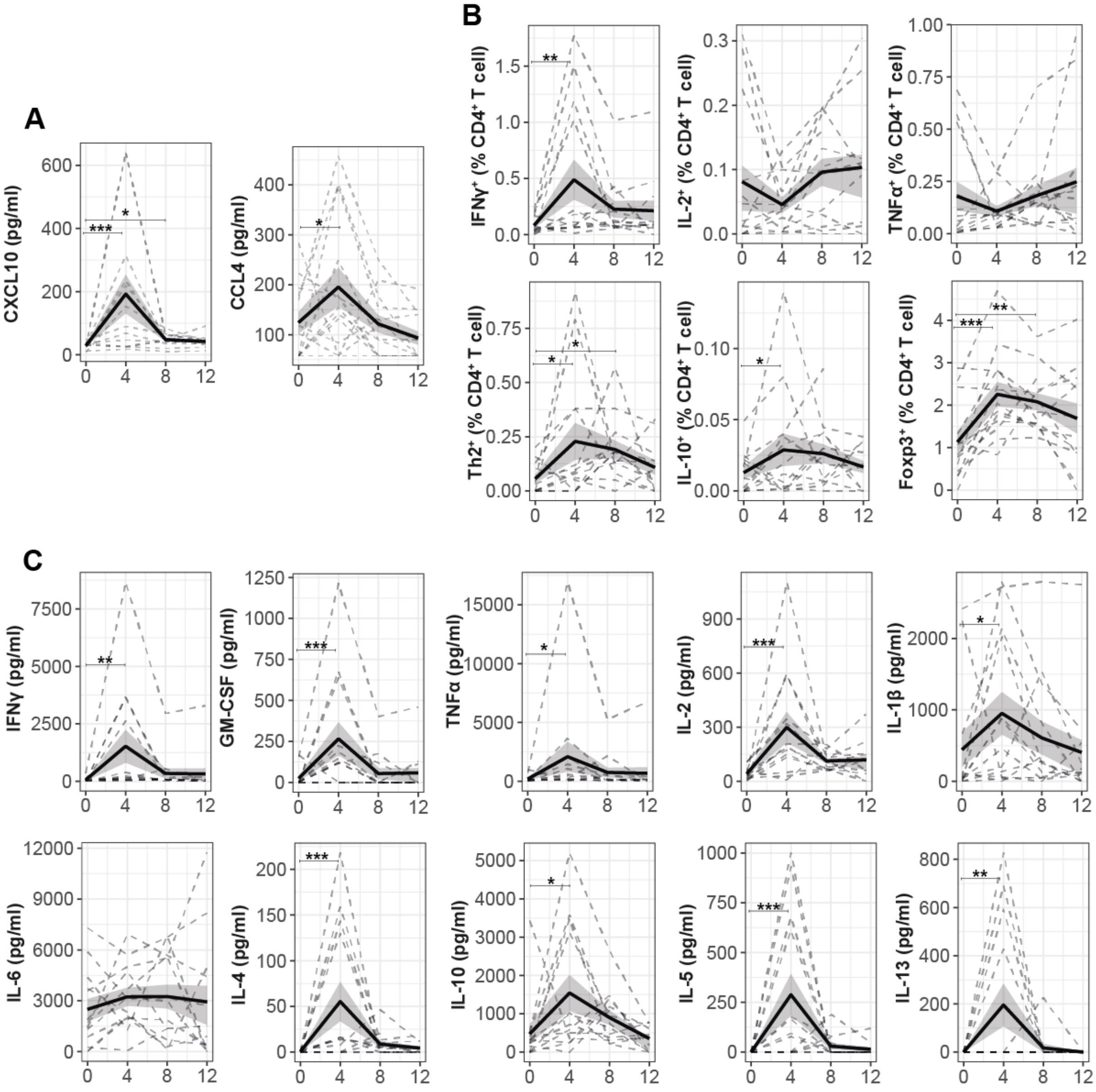

Serum cytokines CXCL10 and CCL4, measured by Luminex multiplex immunoassay **(A)**. PBMCs were stimulated for 24hrs with 50µg/ml AWA or medium before analysis of intracellular cytokines via flow cytometry **(B)** and secreted cytokines via Luminex multiplex immunoassay **(C).** Responses are displayed post subtraction of corresponding medium stimulated values. Dashed lines represent individual participants, solid line the overall mean and the grey ribbon encompassing the standard error of the mean. For plotting, negative values have been shown as 0. Linear mixed models were fitted, with p-values shown on plots estimated using Satterthwaite's method for degrees of freedom. *p<0·05, **p<0·01, ***p<0·001.

Supplementary Fig 6. Individual sPLS-DA prediction analyses

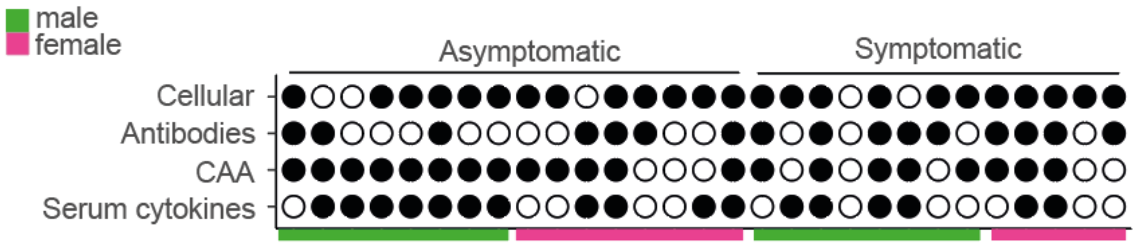

Filled circles indicate a correct prediction and open circles a false prediction of acute schistosomiasis using individual sPLS-DA analyses per row. Individual participants shown in columns, blocks in rows. Model trained on male and female infection data, predictions result of LOO-CV. Tuning was performed to determine the features chosen within each sPLS-DA, with a range of 1-4 features per component.

Supplementary Table 1. Microscopy counts of cercariae in rinse water after exposure

	**10 cercariae (n=3)**	**20 cercariae (n=10)**
**Heads, median (range)**	1 (0-1)	1 (0-1)
**Tails, median (range)**	8 (7-9)	7 (4-13)
**Whole cercariae, median (range)**	0 (0-2)	0 (0-1)
