# Supplementary material for: Safety and infectivity of female cercariae in Schistosoma-naïve, healthy participants: a controlled human Schistosoma mansoni infection study
Source: eBioMedicine. 2023 Oct 12;97:104832. doi: 10.1016/j.ebiom.2023.104832 (PMC10585222; doi:10.1016/j.ebiom.2023.104832)
Supplement: Protocol [file mmc2.pdf]

Establishing a Female-only Controlled Human *Schistosoma mansoni* Infection Model: a safety and dose finding study (CoHSI2)

Version 2.0, 22 January 2021

PROTOCOL TITLE 'Establishing a Female-only Controlled Human *Schistosoma mansoni* Infection Model: a safety and dose finding study'

**Protocol ID**	CoHSI2
**Short title**	Controlled Human *Schistosoma mansoni* Infection
**Version**	2.0
**Date**	22 January 2021
**Principal investigator(s) (in Dutch: hoofdonderzoeker/ uitvoerder)**	**Dr. M. Roestenberg**<br>Email: M.Roestenberg@lumc.nl<br>Tel: 071-5262102
**Investigator**	**Prof. Dr. L.G. Visser**<br>Email: L.G.Visser@lumc.nl<br>Tel: 071-5264838<br><br>**Drs. J.P.R. Koopman**<br>Email: J.P.R.Koopman@lumc.nl<br>**Drs. V.P. Kuiper**<br>Email: V.P.Kuiper@lumc.nl<br>**Drs. M. Hoogerwerf**<br>Email: M.Hoogerwerf@lumc.nl<br><br>Tel: 071-5268702
**Sponsor (in Dutch: verrichter/opdrachtgever)**	Department of Parasitology<br>Leiden University Medical Center<br>**Prof. Dr. M. Yazdanbakhsh**<br>Email: M.Yazdanbakhsh@lumc.nl<br>Tel: 071-5264637
**Data management and trial coordination**	**Mw J.J. Janse, MSc**<br>Email: J.J.Janse<br>Tel: 071-5268702
**Independent expert**	**Dr. M.P. Bauer**<br>M.P.Bauer@lumc.nl<br>071-5262381
**Production site for cercariae**	Department of Parasitology, under supervision of the IGFL (interdivision GMP facility)<br>**Dr. P. Meij**<br>Email: P.Meij@lumc.nl

	Tel: 071-5264177<br><br>**Dr. I.M. Amerongen-Westra**<br>Email: I.M.Westra@lumc.nl<br>Tel: 071-5264179<br><br>**Mw. A. Ozir-Fazalalikhan**<br>Email: A.Ozir-Fazalalikhan@lumc.nl<br>Tel: 071-5265072<br><br>**Ing. J.C. Sijtsma**<br>Email: J.C.Sijtsma@lumc.nl<br>Tel: 071-5268702
**Laboratory sites**	Department of Molecular Cell Biology<br>(Schistosoma antigen detection)<br>**Dr. Ir. P.L.A.M. Corstjens**<br>Email: P.Corstjens@lumc.nl<br>Tel: 071-5269209<br><br>**Dr. G.J. van Dam**<br>Email: G.J.vanDam@lumc.nl<br>Tel: 071-5264400<br><br>**Mw. M. Casacuberta-Partal**<br>Email: M.Casacuberta_Partal@lumc.nl<br>Tel: 071-5268702<br><br>(central laboratory)<br>**Mw. J.C.M. Verhagen**<br>Email: J.C.M.Verhagen@lumc.nl<br>Tel: 071-5261211
**Pharmacy**	(clinical pharmacy)<br>**Mw. C.M. van der Hulst**<br>Email: trials@lumc.nl<br>Tel: 071-5265285

**Safety monitoring committee**	**Dr. P.J.J. van Genderen**<br>Email: PJJvanGenderen@travelclinic.com<br><br>**Dr. Amaya Bustinduy**<br>Amaya.Bustinduy@lshtm.ac.uk<br><br>**Dr. Anna Wajja**<br>Anna.Wajja@mrcuganda.org
**Data Protection Officer**	**LUMC Data Protection Officer**<br>Email: infoavg@lumc.nl

PROTOCOL SIGNATURE SHEET

**Name**	**Signature**	**Date**
**Sponsor and head of department:**<br>***Prof. dr. M. Yazdanbakhsh***		22-1-2021
**Principal Investigator:**<br>***Dr. M. Roestenberg***		25-01-2021

TABLE OF CONTENTS

1. INTRODUCTION AND RATIONALE 11
2. OBJECTIVES 13
3. STUDY DESIGN 14
4. STUDY POPULATION 16
4.1 Population (base) 16
4.2 Inclusion criteria 16
4.3 Exclusion criteria 16
4.4 Sample size calculation 17
5. TREATMENT OF SUBJECTS 18
5.1 Non-investigational product/treatment 18
5.2 Use of co-intervention (if applicable) 18
5.3 Escape medication 18
6. NON-INVESTIGATIONAL PRODUCT: female cercariae 19
6.1 Name and description of non-investigational product(s) 19
6.2 Summary of findings from non-clinical studies 19
6.3 Summary of known and potential risks and benefits 19
6.4 Description and justification of route of administration and dosage 19
6.5 Dosages, dosage modifications and method of administration 19
6.6 Preparation and labelling of Non Investigational Medicinal Product 20
6.7 Drug accountability 20
7. NON-INVESTIGATIONAL PRODUCT: praziquantel 21
7.1 Name and description of non-investigational product(s) 21
7.2 Dosages, dosage modifications and method of administration 21
7.3 Preparation and labelling of Non Investigational Medicinal Product 22
7.4 Drug accountability 22
8. METHODS 23
8.1 Study parameters/endpoints 23
8.1.1 Main study parameter/endpoint 23
8.1.2 Other study parameters/endpoints 23
8.2 Randomisation, blinding and treatment allocation 23
8.3 Study procedures 23
8.4 Withdrawal of individual subjects 27
8.5 Replacement of individual subjects after withdrawal 27
8.6 Follow-up of subjects withdrawn from treatment 27
8.7 Premature termination of the study 29
9. SAFETY REPORTING 30
9.1 Temporary halt for reasons of subject safety 30
9.2 AEs, SAEs and SUSARs 30
9.2.1 Adverse events (AEs) 30
9.2.2 Serious adverse events (SAEs) 30
9.2.3 Suspected unexpected serious adverse reactions (SUSARs) 31

9.2.4 Adverse events data collection 32
9.2.5 Assessment of causality 33
9.3 Annual safety report 34
9.4 Follow-up of adverse events 34
9.5 Safety committee 34
10. STATISTICAL ANALYSIS 35
10.1 Primary study parameter(s) 35
10.1.1 Frequency and severity of adverse events 35
10.1.2 Attack rate per dose group 35
10.2 Other study parameters 35
11. ETHICAL CONSIDERATIONS 36
11.1 Regulation statement 36
11.2 Recruitment and consent 36
11.3 Benefits and risks assessment, group relatedness 36
11.4 Compensation for injury 39
11.5 Incentives 39
12. ADMINISTRATIVE ASPECTS, MONITORING AND PUBLICATION 40
12.1 Handling and storage of data and documents 40
12.2 Monitoring and Quality Assurance 40
12.3 Amendments 40
12.4 Annual progress report 41
12.5 Temporary halt and (prematurely) end of study report 41
12.6 Public disclosure and publication policy 41
13. STRUCTURED RISK ANALYSIS 42
13.1 Potential issues of concern 42
13.2 Synthesis 46
14. REFERENCES 48

LIST OF ABBREVIATIONS AND RELEVANT DEFINITIONS

ABR	General Assessment and Registration form (ABR form), the application form that is required for submission to the accredited Ethics Committee; in Dutch: Algemeen Beoordelings- en Registratieformulier (ABR-formulier)
AE	Adverse Event
AR	Adverse Reaction
BMI	Body Mass Index
CA	Competent Authority
CAA	Circulating Anodic Antigen
CCMO	Central Committee on Research Involving Human Subjects; in Dutch: Centrale Commissie Mensgebonden Onderzoek
CV	Curriculum Vitae
EU	European Union
GCP	Good Clinical Practice
HBV	Hepatitis B Virus
HCV	Hepatitis C Virus
HIV	Human Immunodeficiency Virus
IB	Investigator’s Brochure
IC	Informed Consent
LUMC	Leiden University Medical Center
METC	Medical research ethics committee (MREC); in Dutch: medisch-ethische toetsingscommissie (METC)
PD	Product Dossier
PZQ	Praziquantel
(S)AE	(Serious) Adverse Event
SM	*Schistosoma mansoni*
SMC	Safety monitoring committee
Sponsor	The sponsor is the party that commissions the organisation or performance of the research, for example a pharmaceutical company, academic hospital, scientific organisation or investigator. A party that provides funding for a study but does not commission it is not regarded as the sponsor, but referred to as a subsidising party.
SUSAR	Suspected Unexpected Serious Adverse Reaction
WMO	Medical Research Involving Human Subjects Act; in Dutch: Wet Medisch-wetenschappelijk Onderzoek met Mensen

SUMMARY

Rationale: Schistosomiasis is a parasitic disease of global importance, for which no vaccine exists. Vaccine candidates are tested for efficacy in large-scale Phase 2 and 3 field trials in *Schistosoma*-endemic areas, where the endpoint is usually the incidence of infection or disease following natural exposure. Such trials therefore require long duration and/or large population sizes in order to obtain a good estimate of the effect size. Conducting controlled, experimental infection studies have been shown to eliminate several drawbacks of the traditional proof-of-efficacy approach. Previously, we have established a male-only controlled human *Schistosoma mansoni* infection model, that proved to be safe and well-tolerated in healthy *Schistosoma*-naïve healthy volunteers. In this study we aim to develop a female-only controlled human *Schistosoma mansoni* infection model that can be used to provide early proof-of-concept data on candidate schistosomiasis vaccines and serve as a platform to study schistosome immune responses. This is of particular relevance as one of the developed schistosomiasis vaccine candidate's target antigens is preferentially expressed on female schistosomes.

Objective:

Primary Objective:

- To investigate the safety, tolerability and attack rate of female *Schistosoma mansoni* cercariae in healthy Schistosome-naïve volunteers

Exploratory Objectives:

- To investigate the kinetics of circulating anodic antigen (CAA) after infection with female *Schistosoma mansoni* cercariae in healthy Schistosome-naïve volunteers
- To investigate immunological, metabolic and microbiome changes after infection with *Schistosoma mansoni* female cercariae
- To explore potential differences in CAA kinetics and immunological responses after infection with male or female *Schistosoma mansoni* cercariae

Study design: Open label, dose escalation intervention study with adaptive design

Study population: Healthy human volunteers, 18-45 years old

Intervention: Groups of 3 or 7 volunteers will be exposed to female cercariae. Depending on the outcome of infection and safety data, the dose will be escalated or additional volunteers will be exposed to the same number of cercariae. Volunteers will visit the clinical trial centre weekly after infection to record adverse events.

Main study parameters/endpoints:

Main study parameter/endpoint:

- Frequency and severity of adverse events after controlled human *Schistosoma mansoni* infection with female cercariae

- The number of female cercariae at which 100% volunteers show detectable *Schistosoma mansoni* circulating anodic antigen

Other study parameters/endpoints:

- Time to positive serum and urine CAA test
- Comparison of the peak serum CAA concentration in different dose groups
- Humoral (antibody) response directed against *Sm* antigens
- Cellular responses directed against *Sm* antigens
- Changes in microbiome after controlled human *Schistosoma mansoni* infection with female *Sm* cercariae

Nature and extent of the burden and risks associated with participation, benefit and group relatedness: Volunteers will be requested to visit the trial centre on a weekly basis for 16 weeks. After this bi-weekly visits will follow until week 20. Final follow up visit will be after one year. Blood and urine sampling will take place at every visit. Nasosorption sampling is performed during the first eight weeks. They will keep a diary to record adverse events during 20 weeks. Volunteers will be dermally exposed to female cercariae once. They may experience adverse events, related to acute schistosomiasis syndrome with fatigue, malaise, and fever. At 8 weeks and 12 weeks after infection, they will be treated with praziquantel to cure the *Schistosoma* infection. Praziquantel is known to potentially give fatigue, gastrointestinal side effects, and dizziness. There is no benefit to participation in the trial.

1. INTRODUCTION AND RATIONALE

Schistosomes are parasitic helminths of the order Trematoda that are transmitted through snails in (sub)tropical areas. Current estimates suggest that 252 million people are infected with schistosomes [1], with approximately two-thirds of the cases caused by *Schistosoma haematobium* (the cause of urogenital schistosomiasis), one-third caused by *Schistosoma mansoni* (the cause of intestinal schistosomiasis) and approximately one percent caused by *Schistosoma japonicum* and *S. mekongi* (intestinal schistosomiasis). Schistosomiasis together with hookworm infection and leismaniasis rank as the leading neglected tropical diseases in terms of disability–adjusted life years [2]. More than 90% of the schistosomiasis cases are found in Africa (*S. haematobium* and *S. mansoni*), with the remainder of cases caused by *S. mansoni* in Brazil and Latin America. *S. japonicum* and *mekongi* are endemic in areas in South-East Asia. Endemicity of *Schistosoma* is focal, and mainly related to the presence of fresh water snails of the genus *Biomphalaria*. Depending on the presence of the snail, schistosomiasis can spread to subtropical areas such as the island of Corsica in Europe.

Within the snail, parasites reproduce asexually and are released as infective larval swimming schistosomes, known as cercaria. The cercariae enter the skin, lose their tail and migrate through the blood stream and lungs reaching the liver. In the liver, schistosomes mature into male and female worms, which pair and release eggs in the mesenteric or bladder venules. Eggs released into the environment through the digestive or urinary tract hatch and infective miracidiae may infect snails, closing the cycle.

The majority of the human pathology from schistosomiasis occurs in high burden infections when the eggs are unable to exit from the human host, causing inflammatory responses, granulomas and fibrosis. These pathological changes are responsible for the long-term pathology such as portal hypertension and hepatosplenomegaly in the case of *S. mansoni,* and haematuria, urinary tract infections, hydronephrosis and kidney failure in *S. haematobium*. Inflammation of the female reproductive tract may cause cervical lesions that are risk factors for acquiring sexually transmitted diseases such as HIV [3].

Current treatment and control of schistosomiasis is dependent on solely one drug, praziquantel (40 mg/kg single dose). Cure rates with this single dose have been reported to vary between 40-90% [4, 5], but these depend largely on the timing after initial infection, on the pre-treatment parasite load and the intensity of reinfection [6, 7]. Repeated praziquantel dosing may thus be required to achieve full cure in those populations. However, mass treatment with this drug does not prevent reinfection and therefore in exposed populations the disease prevalence returns

to its original level within 6 to 8 months following chemotherapy. The development of a vaccine against schistosomiasis would be an essential tool in the control of this important parasitic disease.

Vaccination studies in mice and non-human primates with radiation-attenuated cercariae have provided the strongest proof-of-concept that vaccination against schistosomiasis is feasible [8, 9]. Antigens from stage-specific parasites have thus been identified as vaccine candidates, aiming to prevent infection or reduce worm burden. The pathway for WHO endorsement of these candidate vaccines would be to generate at least a 40% reduction in worm burdens (http://who.int/immunization/research/meetings_workshops/Schistosomiasis_VaccineRD_Sept2014.pdf).

Typically, these vaccine candidates are tested for proof-of-efficacy in large-scale Phase 2 and 3 field trials in *Schistosoma*-endemic areas, where the endpoint is usually the incidence of infection as detected by egg production, worm antigen or disease following natural infection. To obtain a good estimate of the effect size, trials of long duration and/or large population sizes are necessary. Conducting controlled, experimental infection studies has been shown to eliminate several drawbacks of the traditional proof-of-efficacy approach and has been well-established as a model for downselecting vaccine candidates for malaria, dengue and influenza [10].

In the first controlled human *Schistosoma mansoni* Infection (CoHSI) study that took place between 2016-2018, we successfully developed a male-only controlled human *Schistosoma mansoni* infection model (trial name: CoHSI1 study). In total 17 healthy volunteers were exposed to increasing doses of male cercariae. Controlled infection with 20 male *Schistosoma mansoni* cercariae was safe and well-tolerated, and had a high attack rate (82%) [11]. In analogy, this subsequent study aims to establish a female-only controlled human *Schistosoma mansoni* infection (CoHSI) model.

The sex of the worms has been hypothesised to differentially impact host immune responses. In contrast to males that trigger strong innate immune responses, females are found to dampen innate immune reactions resulting in improved parasite survival [12]. This makes female worms an important vaccine target. Not only would the establishment of a female-only CoHSI model allow exploration of sex-specific immune responses, but also it may be used to provide early proof-of-concept data on candidate schistosomiasis vaccines. This is of particular relevance as one of the developed schistosomiasis vaccine candidate's target antigens is preferentially expressed on female schistosomes.

2. OBJECTIVES

Primary Objective:

- To investigate the safety, tolerability and attack rate of female *Schistosoma mansoni* cercariae in healthy Schistosome-naïve volunteers

Exploratory Objectives:

- To investigate the kinetics of circulating anodic antigen (CAA) after infection with female *Schistosoma mansoni* cercariae in healthy Schistosome-naïve volunteers
- To investigate immunological, metabolic and microbiome changes after infection with *Schistosoma mansoni* female cercariae
- To explore potential differences in CAA kinetics and immunological responses after infection with male or female *Schistosoma mansoni* cercariae

3. STUDY DESIGN

This study is an open label, dose escalation intervention trial with an adaptive design.

Volunteers will be exposed to predefined doses of cercariae in groups of three and subsequently seven volunteers. Dose escalation will be performed according to the schedule depicted in figure 1. If all three volunteers in one group show detectable circulating anodic antigen in serum, seven additional volunteers will be exposed to the same dose. If not all volunteers in one dose group become infected, the next group of three volunteers will be exposed to a higher dose. The highest dose is 60 cercariae. If one or more volunteers from group A of 60 cercariae have a positive CAA test, another seven volunteers will also be exposed to this dose (group B). If both group A and group B volunteers (total 10) were exposed to the same dose, the study will stop.

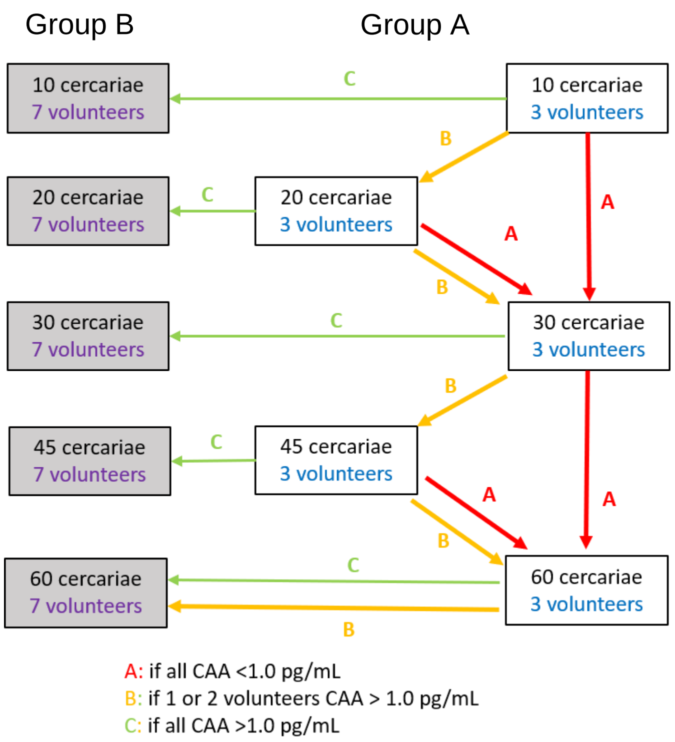

Figure 1. Dose schedule. In case all volunteers become positive for Schistosoma circulating anodic antigen, the additional volunteers will be exposed to the same cercariae dose (green arrows, continue from group A to B). In case not all volunteers become positive for Schistosoma circulating anodic antigen, the dose will escalate (red or yellow arrows).

In the clinical study performed earlier we exposed four groups to male cercariae. The starting dose for the first group (A1) was also 10 cercariae. After dose-escalation to 30 cercariae for the second group (A2), we decided to dose de-escalate and expose the third group (A3) to 20 cercariae because of the burden of severe adverse events related to acute schistosomiasis syndrome in one of the volunteers (please refer to paragraph 11.3 or the Product Dossier section 2.3.2: Human exposure" for more information). The last cohort was then also exposed to 20 cercariae (B). We have thus adjusted the dose escalation schedule to allow for slower escalation if needed.

In addition, we have included cercarial doses that are higher than those used in the male cercariae infection study. This has been done for two reasons: 1) rodent experiments suggest that female cercariae may be less likely to establish infection as worm counts rendered fewer numbers in single-sex female infected animals when compared to single sex male infected animals; and 2) because female worms do not fully mature if they are unable to mate, CAA levels are expected to be lower than in mixed or single-sex male infections.

All volunteers included in the trial will be followed as out-patients for one year. They will be followed on a weekly basis starting at week 1 after infection until 16 weeks after infection. At week 18, 20 and one year after infection the final visits will take place. All volunteers will be treated with praziquantel (PZQ) 60 mg/kg at 8 weeks after infection and at 12 weeks after infection (Figure 2).

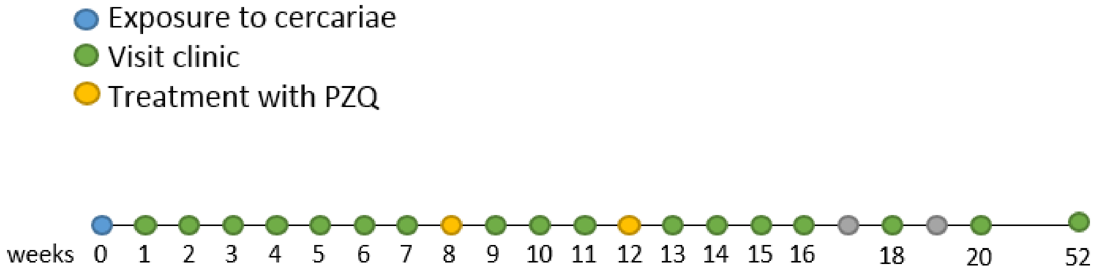

Figure 2. Trial schematic for CoHSI2 study

4. STUDY POPULATION

4.1 Population (base)

Healthy subjects, male or female, aged 18-45 years old will be included in the study from the general population.

4.2 Inclusion criteria

In order to be eligible to participate in this study, a subject must meet all of the following criteria:

1. Subject is aged ≥ 18 and ≤ 45 years and in good health.
2. Subject has adequate understanding of the procedures of the study and agrees to abide strictly thereby.
3. Subject is able to communicate well with the investigator, is available to attend all study visits.
4. Subject will remain within Europe (excluding Corsica) during the study period and is reachable by mobile telephone from week 3 to week 8 of the study period.
5. Subject agrees to refrain from blood donation to Sanquin or for other purposes throughout the study period.
6. For female subjects: subject agrees to use adequate contraception and not to breastfeed for the duration of study.
7. Subject has signed informed consent.

4.3 Exclusion criteria

A potential subject who meets any of the following criteria will be excluded from participation in this study:

1. Any history, or evidence at screening, of clinically significant symptoms, physical signs or abnormal laboratory values suggestive of systemic conditions, such as cardiovascular, pulmonary, renal, hepatic, neurological, dermatological, endocrine, malignant, haematological, infectious, immune-deficient, psychiatric and other disorders, which could compromise the health of the volunteer during the study or interfere with the interpretation of the study results. These include, but are not limited to, any of the following:
    - body weight <50 kg or Body Mass Index (BMI) <18.0 or >30.0 kg/m^2 at screening;
    - positive HIV, HBV or HCV screening tests;
    - the use of immune modifying drugs within three months prior to study onset (inhaled and topical corticosteroids and oral anti-histamines exempted) or expected use of such during the study period;
    - history of malignancy of any organ system (other than localized basal cell carcinoma of the skin), treated or untreated, within the past 5 years;
    - any history of treatment for severe psychiatric disease by a psychiatrist in the past year;

- history of drug or alcohol abuse interfering with normal social function in the period of one year prior to study onset.

2. The chronic use of any drug known to interact with praziquantel, artesunate or lumefantrine metabolism (e.g. phenytoïn, carbamazepine, phenobarbital, primidon, dexamethason, rifampicine, cimetidine, flecaïnide, metoprolol, imipramine, amitriptyline, clomipramine, class IA and III anti-arrythmics, antipsychotics, antidepressants, macrolides, fluorchinolones, imidazole- and triazole antimycotics, antihistamines) Because lumefantrine may cause extension of QT-time, chronic use of drugs with effect on QT interval are excluded from the study.
3. For female subjects: positive urine pregnancy test at screening.
4. Any history of schistosomiasis or treatment for schistosomiasis.
5. Positive serology for schistosomiasis or elevated serum CAA at screening.
6. Known hypersensitivity to or contra-indications (including co-medication) for use of praziquantel, artesunate or lumefantrine.
7. Being an employee or student of the department of parasitology or infectious diseases of the LUMC.

4.4 Sample size calculation

Subject to safety and tolerability we aim in these studies to achieve a 100% infection rate in 10 subjects. At a lower infection rate of 80% (for example) this sample size will estimate attack rate with precision of approximately +/-22.5%; i.e. ruling out an attack rate of less than 50%. For future vaccine proof-of-concept efficacy studies, small groups of roughly 10 subjects are generally preferred, allowing for detection of roughly ~50% protective vaccines with a power of 80% (alpha <0.05, 2-tailed). A 100% attack rate in the infectivity control group (i.e. group exposed to pathogen, but not receiving the vaccine) is required to achieve this power. With a 80% attack rate and equal number of subjects (n=10), the power to detect an estimated 50% efficacy is reduced to 63%. Therefore, we aim in this study to achieve a 100% attack rate in 10 subjects.

5. TREATMENT OF SUBJECTS

5.1 Non-investigational product/treatment

Volunteers will be exposed to a predefined dose of female cercariae according to the schedule depicted in figure 1, by applying female cercariae to the intact skin of the forearm for 30 minutes. Cercariae will be applied to the skin in 0.5-1.0 ml Bar-le-duc water. Every volunteer will be exposed to cercariae once.

5.2 Use of co-intervention

Severe itching at the site of entry of the larvae (swimmer's itch) may be treated with triamcinoloncream 0.5% (applied twice daily) according to local clinical practice [13].
In case of symptoms of acute schistosomiasis syndrome (Katayama fever), subjects will receive symptomatic treatment such as paracetamol or, in case of severe symptoms lasting more than 48 hours, NSAIDs or oral prednisone in doses up to 30 mg. Dosing and administration of these drugs or any other symptomatic drugs will be determined according to severity and duration of the adverse events. Female volunteers are requested to use adequate contraception. There are no dietary requirements.

5.3 Escape medication

The trial physician may decide to abrogate the study for individual subjects according to the criteria described in section 8.4. Because early treatment of schistosomiasis infection has been shown to aggravate clinical symptoms in reported cases, the advantages and disadvantages of such escape medication should be carefully weighed. Escape medication should, in symptomatic cases, be accompanied by symptomatic treatment with corticosteroids.
Escape medication to abrogate the study for individual volunteers before week 8 of the study will be artemether/lumefantrine in a standard 6-dose treatment regimen over 3 days. Volunteers with early abrogation of infection are also treated with praziquantel 60 mg/kg at week 8.

6. NON-INVESTIGATIONAL PRODUCT: female cercariae

6.1 Name and description of non-investigational product(s)

Volunteers will be exposed to a predefined dose of female cercariae according to the schedule depicted in figure 1, by applying female cercariae to the intact skin of the forearm for 30 minutes. Every volunteer will be subjected to one exposure to cercariae. For details on the production and quality control of these female cercariae, please refer to the Product Dossier section 2.1.P.3 “Manufacture” and 2.1.P.5 “Control of the cercarial product”.

6.2 Summary of findings from non-clinical studies

Please refer to the Product Dossier section 2.2 “Non-clinical data and toxicology”.

6.3 Summary of known and potential risks and benefits

Please refer to the Product Dossier section 2.4 “Overall risk and benefit assessment”. In addition, a structured risk analysis is given in chapter 13 of this protocol.

6.4 Description and justification of route of administration and dosage

Female cercariae will be allowed to penetrate the skin of human volunteers by applying 0.5-1 ml of Bar-le-duc water containing the specified number of cercariae on the skin for 30 minutes. This route has been chosen because it is the natural route of infection and complications of the site of entry other than swimmer’s itch have not been described. From non-human primate and rodent experience we expect roughly 5-70% of cercariae to develop into mature worms after penetration of the skin. Also from rodent experiments we expect that female cercariae will not fully mature because they are not able to mate. As such, they will produce less CAA as compared to the same number of mixed-sex infections. We extrapolated that we would need roughly 5 mature female worms to be able to potentially detect circulating CAA. Because not all cercariae may penetrate the skin we chose 10 cercariae as the minimal dose for controlled human infection that may possibly be detectable in a number of volunteers, which is the same starting dose as for the single-sex infection with male *Sm.* The dose-escalation scheme as shown is figure 1 takes into account that 1) fewer female worms may reach maturity and 2) CAA may be lower than in mixed-sex or single-sex male infections.

6.5 Dosages, dosage modifications and method of administration

Doses of cercariae will be prepared by manual counting and checked by a second technician. Cercariae will be administered by natural penetration of the skin in 0.5-1 ml of Bar-le-duc water.

6.6 Preparation and labelling of Non Investigational Medicinal Product

Cercariae will be prepared in the A1 well of a 24-well plate. The plate will be labelled with the following sticker, which will be completed at the time of preparation:

FEMALE CERCARIAE for the purpose of CoHSI<br><br>Trial identifier: CoHSI2<br>Snail identifier:<br>Total number:<br>Well:<br>Date:<br><br>Time end shedding:<br>Time plate ready:<br>Do not use after: (time)<br><br>Name technicians:<br>Signatures:<br>For the purpose of medical research only

6.7 Drug accountability

After controlled human schistosomiasis infection, the number of tails, heads and intact cercariae will be counted under the microscope (of the A1 well of the 24-well plate, of the water on the forearm of the volunteer, and of the rinsing water used for each volunteer). Numbers will be noted in the clinical trial database. All equipment used is disposable and will be destroyed according to biosafety level 2 regulations.

7. NON-INVESTIGATIONAL PRODUCT: praziquantel

7.1 Name and description of non-investigational product(s)

Praziquantel

All volunteers will be treated with 60 mg/kg praziquantel at week 8 and week 12 after infection. Praziquantel is the only drug registered for the treatment of schistosomiasis. Cure rates with a single dose of 40 mg/kg has been reported to vary between 40-90% [4, 5] in endemic areas, however in travelers the efficacy appears lower [14] which may be caused by lower infection intensity or differential host-immune responses. Therefore, repeated praziquantel doses may be required to achieve full cure. A common side effect of praziquantel (>10%) is fatigue. Other less common side effects are abdominal discomfort, nausea, and dizziness. For a summary of findings from non-clinical and clinical studies, please refer to the “bijsluiter” (SPC Praziquantel). Praziquantel has been described to temporarily impair skilled tasks, and thus volunteers treated with praziquantel will be advised not to drive a car or operate machinery on the day of treatment with praziquantel and the day thereafter [15]. They are instructed to take praziquantel after a meal preferably containing high-fat, and high carbohydrate content [16].

7.2 Dosages, dosage modifications and method of administration

The dose administered will be 60 mg/kg praziquantel, split into two doses during one day. This dose is higher than the 40 mg/kg dose recommended by WHO for preventive chemotherapy in endemic settings. We chose the 60 mg/kg dose because of two reasons. Firstly, meta-analysis by Zwang *et al*. suggested that cure rates with single-dose 60 mg/kg were slightly higher than with single-dose 40 mg/kg (84.6% vs. 76.7%, respectively), however confidence intervals around the estimates were overlapping [17].Secondly, in mice the EC^{50} for single-sex female infections appeared to be higher than for single-sex male or mixed-sex infections [18].

In addition to treatment at week 8, all volunteers will receive a second round of treatment at week 12 to ensure that any previously immature worms, that were less susceptible to praziquantel at week 8 [18], are also killed.

Although more recently, WHO advises a single-dose in MDA to improve uptake, splitting the dose in our study (i.e. two doses during one day) may reduce risk of side effects. Praziquantel will be taken after a meal.

7.3 Preparation and labelling of Non Investigational Medicinal Product

Praziquantel will be prepared and labelled for individual volunteers according to the standard procedures in the treatment of schistosomiasis. Each volunteer will receive exactly the calculated number of tablets calculated on weight basis:

**60 mg/kg dose**	
**Weight (kg)**	**Number of tablets (600 mg each)**
48-52	5
53-57	5½
58-62	6
63-67	6½
68-72	7
73-77	7½
78-82	8
83-87	8½
88-92	9
93-97	9½

7.4 Drug accountability

Praziquantel tablets are dispensed by the hospital pharmacy in containers with 6 tablets. The exact number of tablets of praziquantel will be distributed to the volunteers by the clinical investigator. Remaining tablets will be returned to the pharmacy for destruction according to appropriate regulations. A drug accountability log will be kept through signature lists at the trial site. These records will include:

- volunteer identification number;
- number of tablets dispensed and remaining;
- signature of the clinical investigator dispensing the tablets.

8. METHODS

8.1 Study parameters/endpoints

8.1.1 Main study parameter/endpoint

- Frequency and severity of adverse events after controlled human *Schistosoma mansoni* infection with female cercariae
- The number of female cercariae at which 100% volunteers show detectable *Schistosoma mansoni* circulating anodic antigen

8.1.2 Other study parameters/endpoints

- Time to positive serum and urine CAA test
- Comparison of the peak serum CAA concentration in different dose groups
- Humoral (antibody) response directed against *Sm* antigens
- Cellular responses directed against *Sm* antigens
- Changes in microbiome after controlled human *Schistosoma mansoni* infection with female *Sm* cercariae

8.2 Randomisation, blinding and treatment allocation

The trial will be an open label trial, so there will be no randomisation or blinding procedure.

8.3 Study procedures

Recruitment of volunteers

Advertisements will be placed in prominent places on the university and hospital campuses and other public places as well as on the internet and intranet site of the LUMC. Furthermore, a Facebook page showing the advertisement text will be designed to inform people about the trial. The study will be advertised on social media. This brief advertisement will indicate a telephone number to call and an e-mail address for contact to request further information. It will furthermore indicate a website (www.vaccinonderzoek.nl) which contains a form to request further information. We will also reach out to people via email to people who have subscribed to our newsletter.
All volunteers with interest in participation in the study will be advised to join one of the information meetings and will receive the study documents (the information sheet, the application form and the insurance text about the study. If they are still interested after the meeting or after reading the documents, an appointment for a screening visit will be made.

The screening visit will be planned at least 72 hours after the subject receives the information sheet and informed consent form.

Screening visit

During the screening visit, volunteers are first given the opportunities to address any study related questions after which they will be asked to sign the informed consent form if they still want to participate. All volunteers must consent to an HIV, hepatitis B, hepatitis C serological screening, urine toxicology and for females a pregnancy test at screening. A copy of the informed consent form will be given to the volunteer. Subsequently volunteers are asked to complete an application form which includes a questionnaire regarding their health. The questionnaire answers will be discussed and inclusion and exclusion criteria will be checked. The possibility of withdrawal from the infection study, at any time and without any declaration of the reason as well as the resulting necessary follow-up visits for safety will be pointed out to the volunteers. All volunteers will be asked to supply a phone number of a person who may be contacted in case of emergency.

A physical examination will be performed, including the examination of general appearance, skin, neck, eyes, throat, lungs, heart, abdomen, back and extremities, and a routine vascular and neurological examination. Vital signs (tympanic temperature, blood pressure (BP) and pulse) will be measured.

Height (cm) and body weight (to the nearest kilogram [kg] in indoor clothing, but without shoes) will be measured at the screening visit. Body mass index (BMI) will be calculated using the following formula: BMI = Body weight (kg) / $[\text{height (m)}]^2$.

Infection with female *Sm* cercariae

At the day of exposure to female *Sm* cercariae, volunteers will visit the clinical trial centre in the morning for a final check of in- and exclusion criteria, a focussed physical exam and vital signs. Blood will be drawn for baseline assessments and safety lab will be checked. In the afternoon, exposure to female *Sm* cercariae will be performed at the laboratory of the parasitology department (P4) within the LUMC building 1. Female cercariae will be allowed to penetrate the skin of human volunteers by applying 0.5-1ml of Bar-le-duc water containing the specified number of cercariae on the skin for 30 minutes.

Exposures will be performed by one of the clinical investigators. A nurse or second investigator will be present to check study procedures. Volunteers will be observed for at least 30 minutes after the exposure.

Follow-up after infection

Volunteers will visit the trial centre for screening, then at the day of infection and every week starting from 1 week after infection until 16 weeks after infection. After week 16, biweekly follow-ups will follow until week 20. Final visit will be performed at 1 year after infection. At all follow up visits, adverse events will be recorded, volunteers will undergo a blood draw by venepuncture and will provide a urine sample. At every visit the tympanic temperature will be checked (until week 12). A focused physical examination will be performed if deemed necessary by the trial physician. Volunteers are requested to keep a diary from the day of infection until 20 weeks, in which they record adverse events. A clinical trial physician will be available by mobile phone 24/7 during the 20 weeks clinical trial period. Volunteers will be instructed to report to the clinical trial physician in case of any grade 2 (moderate) or grade 3 (severe) adverse events to ensure early detection and possible treatment of symptoms of acute Schistosomiasis infection (see paragraph 9.2.4 Adverse events data collection for more detailed information).
Additional diagnostics (including serum CAA tests) can be performed on discretion of the trial physician at any time if it is deemed necessary for the safety of the study volunteers.

Treatment with praziquantel

Volunteers will be treated with 60 mg/kg praziquantel at week 8 and at week 12. The treatment will consist of a prespecified number praziquantel 600 mg tablets according to the table in section 7.3. In addition to specific treatment with praziquantel, symptomatic treatment will be administered at the discretion of the attending physician.
Volunteers will not be admitted to the hospital during this study, unless the study doctors or the safety monitoring committee deem it necessary.

Safety laboratory evaluation

During the study, blood samples will be drawn to assess safety of infection. The blood sampling schedule is depicted in the flowchart (figure 3). Safety analysis include: complete blood count (including automated differential count of white blood cells), erythrocyte sedimentation rate, creatinine, potassium, bilirubin, AF, gGT, AST, ALT. Biological safety parameters will be measured on plasma or serum samples at the central laboratory of the LUMC. Assessment of successful infection will be performed by serum CAA measurements at the department of Molecular Cell Biology of the LUMC at 8 weeks after exposure to cercariae. Another assessment of CAA levels will be performed at week 20 to confirm cure. If volunteers are not cured after two doses of praziquantel, a third round of treatment will be given and its effect on CAA levels monitored. *Schistosoma mansoni* antigen detection tests will be performed retrospectively on all samples, unless

the investigator, safety monitoring committee or accredited METC requests specific testing of certain samples for clinical judgment of adverse events.
In the case where a safety laboratory assessment is outside the reference range for the laboratory, a decision regarding whether the result is of clinical significance or not shall be made by the investigator and shall be based, in part, upon the nature and degree of the observed abnormality. The assessment may first be repeated for confirmation. In all cases, the investigator will document in the source documents, the clinical considerations (i.e., result was/was not clinically significant and/or medically relevant) in allowing or disallowing the subject to continue in the study. Since elevated eosinophils are an expected result of this study this measure will not be marked as abnormal in the eCRF.
The maximum amount of blood drawn is 560 ml for the 20 weeks study period.

Serology, toxicology, pregnancy test

A serum sample will be obtained at screening to test for the presence of HIV antigen and antibodies, hepatitis B surface antigen, anti-Hepatitis C antibodies.
A midstream urine sample (approx. 30 ml) will be obtained at the screening visit, in order to avoid contamination with epithelial cells and sediments, to allow proper assessments of amphetamines, cocaine and a commercially available hCG urine test.

Source data

All data collected by the investigator is reported in electronic case report forms. These forms, together with the investigator's notes are considered source data. In case of adverse events/reactions resulting in a medical consultation or hospitalization a medical file can be made. In this case the medical file will be considered as the source data. The diaries, produced by the study volunteers are also considered source data. They will be kept as source documents in the investigator clinical file.

Recording of adverse events

Signs and symptoms will be recorded at all visits and whenever a trial volunteer reports signs or symptoms to the trial physician between visits. The following signs and symptoms will be solicited at all visits: itching, fever (by examination), rash, urticaria, headache, fatigue, malaise, coughing, myalgia, arthralgia, night sweats, back pain, anorexia, nausea, vomiting, abdominal pain, diarrhoea.

Exploratory immunology and metabolomics

The overall objective is to find immunological or metabolic markers that associate with female *Schistosoma mansoni* infection. The sampling schedule is depicted in the flow chart below (figure 3).
Humoral assessment will include antibody assays by immunofluorescence, ELISAs and arrays for specific *Schistosoma mansoni* proteins or glycans.
Cellular assessment of parasite-specific (subset) T cell responses, will be conducted by multi-parameter flow cytometry, cyTOF and ELISPOT assays with or without using Sm-specific *in vitro* stimulation.
The metabolic profile of serum and urine samples will be evaluated at timepoints before, during and after infection.
An analysis of faecal samples to investigate the gut microbiome will be performed at 7 timepoints during the trial.
During the first 8 weeks of infection, nasal lining fluid will be collected by 2 min nasosorption in a matrix strip (Nasosorption™, Hunt Developments) and frozen at -80°C. This is a non-invasive procedure. After thawing, various immune parameters can be measured by multiplex platforms, like Luminex of Proseek (Olink). Nasal metabolomics can also be performed.

8.4 Withdrawal of individual subjects

Subjects can leave the study at any time for any reason if they wish to do so without any consequences. However, for safety purposes volunteers will be required to complete some follow-up visits to monitor treatment. The investigator can decide to withdraw a subject from the study for urgent medical reasons.

8.5 Replacement of individual subjects after withdrawal

Subjects will be replaced after withdrawal, if a subject withdraws from group A of 3 volunteers. In that case, the second group from the same dose will increase to 8 volunteers.

8.6 Follow-up of subjects withdrawn from treatment

If a subject fails to appear for a follow-up examination, extensive effort (i.e. documented phone calls and certified mail) will be undertaken to locate or recall him or at least to determine his health status. These efforts will be documented in the source documents. In the event that a volunteer discontinues the study for any reason, he/she will be urged to complete all safety follow-up, including treatment with praziquantel.

Figure 3. Flow chart

	**Screening**	**Challenge**	**Follow up**																		
Deviation			3 days																		4 weeks
Visit		V1	V2	V3	V4	V5	V6	V7	V8	V9	V10	V11	V12	V13	V14	V15	V16	V17	V18	V19	V20
**Week**		**0**	**1**	**2**	**3**	**4**	**5**	**6**	**7**	**8**	**9**	**10**	**11**	**12**	**13**	**14**	**15**	**16**	**18**	**20**	**52**
Obtain informed consent	X																				
Clinical evaluation	X	X	X	X	X	X	X	X	X	X	X	X	X	X	X	X	X	X	X	X	X
Exposure to cercariae		X																			
Praziquantel (60 mg/kg)										X				X							
Eosinophils				X		X	X	X	X			X		X		X		X	X	X	X
Safety tests	X[1,2]	X[2]								X[2]											
Urine pregnancy test for female volunteers	X	X																			
UCP CAA (serum)	X	X	X	X	X	X	X	X	X	X	X	X	X	X	X	X	X	X	X	X	X
UCP CAA (urine)		X	X	X	X	X	X	X	X	X	X	X	X	X	X	X	X	X	X	X	X
Schistosomiasis serology: worm IFA	X					X	X	X	X	X				X				X		X	X
Schistosomiasis serology: egg ELISA	X					X				X				X				X		X	X
Schistosomiasis PCR eggs										X											
Immunological assays (antibody and cellular)		X				X				X				X				X		X	X
Metabolomics	X	X	X	X	X	X	X	X	X	X	X	X		X		X		X	X	X	X
Microbiome		X				X				X				X				X		X	X
Nasosorption		X	X	X	X	X	X	X	X	X											

1: HIV, HBV, HCV, urine cocaine and amphetamines
2: automated CBC, ESR, creatinine, BUN, sodium, potassium, bilirubin, AF, γGT, AST, ALT, glucose

8.7 Premature termination of the study

The study may be discontinued for one of the following reasons:

- a serious adverse event which is possibly, probably or definitely related to the infection with female *Schistosoma mansoni* cercariae;
- >50% of subjects in a dose group (more than 1 out of 3 from group A or more than 5 out of 10 from groups A and B) experience a grade 3 adverse event, possibly, probably or definitely related to infection with female *Schistosoma mansoni* cercariae and persisting at grade 3 for >48 hours during the 20 weeks follow-up;
- on advice from the SMC;
- on advice from the investigator, pending discussion with the SMC;
- on advice from the accredited METC.

9. SAFETY REPORTING

9.1 Temporary halt for reasons of subject safety

In accordance to section 10, subsection 4, of the WMO, the sponsor will suspend the study if there is sufficient ground that continuation of the study will jeopardise subject health or safety. The sponsor/investigator will notify the accredited METC without undue delay of a temporary halt including the reason for such an action. The study will be suspended pending a further positive decision by the accredited METC. The investigator will take care that all subjects are kept informed.

9.2 AEs, SAEs and SUSARs

9.2.1 Adverse events (AEs)

Adverse events are defined as any undesirable experience occurring to a subject during the study, whether or not considered related to infection with female *Schistosoma mansoni* infection. All adverse events reported spontaneously by the subject or observed by the investigator or his staff will be recorded.

9.2.2 Serious adverse events (SAEs)

A serious adverse event is any untoward medical occurrence or effect that

- results in death;
- is life threatening (at the time of the event);
- requires hospitalisation or prolongation of existing inpatients' hospitalisation;
- results in persistent or significant disability or incapacity;
- is a congenital anomaly or birth defect; or
- any other important medical event that did not result in any of the outcomes listed above due to medical or surgical intervention but could have been based upon appropriate judgement by the investigator.

An elective hospital admission will not be considered as a serious adverse event.

A grade 3 AE will not be considered a SAE per se.

The investigator will report all SAEs to the sponsor without undue delay after obtaining knowledge of the events. The sponsor/investigator will report the SAEs through the web portal *ToetsingOnline* to the accredited METC that approved the protocol, within 7 days of first knowledge for SAEs that result in death or are life threatening followed by a period of maximum of 8 days to complete the initial

preliminary report. All other SAEs will be reported within a period of maximum 15 days after the sponsor has first knowledge of the serious adverse events.

9.2.3 Suspected unexpected serious adverse reactions (SUSARs)

Adverse reactions are all untoward and unintended responses to an investigational product related to any dose administered.

Unexpected adverse reactions are SUSARs if the following three conditions are met:

1. the event must be serious (see chapter 9.2.2);
2. there must be a certain degree of probability that the event is a harmful and an undesirable reaction to the medicinal product under investigation, regardless of the administered dose;
3. the adverse reaction must be unexpected, that is to say, the nature and severity of the adverse reaction are not in agreement with the product information as recorded in:
   - Summary of Product Characteristics (SPC) for an authorised medicinal product;
   - Investigator's Brochure for an unauthorised medicinal product.

The sponsor will report expedited the following SUSARs through the web portal *ToetsingOnline* to the METC:

- SUSARs that have arisen in the clinical trial that was assessed by the METC;
- SUSARs that have arisen in other clinical trials of the same sponsor and with the same medicinal product, and that could have consequences for the safety of the subjects involved in the clinical trial that was assessed by the METC.

The remaining SUSARs are recorded in an overview list (line-listing) that will be submitted once every half year to the METC. This line-listing provides an overview of all SUSARs from the study medicine, accompanied by a brief report highlighting the main points of concern.

The expedited reporting of SUSARs through the web portal Eudravigilance or ToetsingOnline is sufficient as notification to the competent authority.

The sponsor/investigator will report expedited all SUSARs to the competent authorities in other Member States, according to the requirements of the Member States.

The expedited reporting will occur not later than 15 days after the sponsor has first knowledge of the adverse reactions. For fatal or life threatening cases the term will be maximal 7 days for a preliminary report with another 8 days for completion of the report.

9.2.4 Adverse events data collection

Safety assessments will be performed, and recorded by the investigators. All adverse events/reactions (solicited and unsolicited), observed by the investigators or by the subject, will be accurately documented in the case report form by the investigators. For each event/reaction the following details will be recorded:

1. Description of the event(s)/reaction(s)
2. Date and time of occurrence
3. Duration
4. Intensity
5. Relationship with the intervention
6. Action taken, including treatment
7. Outcome

Symptoms will be ranked as (1) mild, (2) moderate, or (3) severe, depending on their intensity according to the following scale:

- Mild (grade 1): awareness of symptoms that are easily tolerated and do not interfere with usual daily activity
- Moderate (grade 2): discomfort that interferes with or limits usual daily activity
- Severe (grade 3): disabling, with subsequent inability to perform usual daily activity, resulting in absence or required bed rest

For fever, the following scale will be used:

- Mild (grade 1): 37.5 - 38.0°C
- Moderate (grade 2): > 38.0 to 39.0°C
- Severe (grade 3): > 39.0°C

Temperature will be measure tympanically.

If an AE changes in frequency or intensity during the specified reporting period, these changes will be documented in the eCFR. The highest severity grade will be noted in the eCFR.

When an AE/SAE occurs, it is the responsibility of the investigators to review all documentation (e.g. hospital progress notes, laboratory, and diagnostics reports) related to the event. The investigators will then record all relevant information regarding an AE/SAE on the eCRF or SAE Report Form, respectively.

The investigator will attempt to establish a diagnosis of the event based on signs, symptoms, and/or other clinical information. In such cases, the diagnosis should be documented as the AE/SAE and not the individual signs/symptoms.

9.2.5 Assessment of causality

The investigators are obligated to assess the relationship between study procedures and the occurrence of each AE/SAE. The investigators will use clinical judgment to determine the relationship. Alternative causes, such as natural history of the underlying diseases, concomitant therapy, other risk factors and the temporal relationship of the event to the challenge will be considered and investigated. The relationship of the adverse event with the study procedures will be categorized as:

- Not related: A relationship to the administration of the *Sm* female cercariae cannot be reasonably established; another etiology is known to have caused the adverse event or is highly likely to have caused it.
- Unlikely: A relationship to the administration of *Sm* female cercariae is unlikely; however, it cannot be ruled out.
- Possible: There is a potential association between the event and administration of the *Sm* female cercariae; however, there is an alternative etiology that is a more likely.
- Probable: Administration of the *Sm* female cercariae is the most likely cause; however, there are alternative reasonable explanations, even though less likely.
- Definitely: Administration of the *Sm* female cercariae is the cause; another etiology causing the adverse event is not known.

When a regulatory authority requests a binary classification (related vs. unrelated), definitely, probably and possibly related are considered to be “related,” while not related and unlikely related are considered to be “unrelated.” Thus, an intervention-related AE refers to an AE for which there is a possible, probable or definite relationship to the study intervention. The investigator will use clinical judgment to determine the relationship.

9.3 Annual safety report

In addition to the expedited reporting of SUSARs, the sponsor will submit, once a year throughout the clinical trial, a safety report to the accredited METC.

This safety report consists of:

- a list of all suspected (unexpected or expected) serious adverse reactions, along with an aggregated summary table of all reported serious adverse reactions, ordered by organ system, per study;
- a report concerning the safety of the subjects, consisting of a complete safety analysis and an evaluation of the balance between the efficacy and the harmfulness of the medicine under investigation.

9.4 Follow-up of adverse events

All AEs will be followed until they have abated, or until a stable situation has been reached. Depending on the event, follow up may require additional tests or medical procedures as indicated, and/or referral to the general physician or a medical specialist. SAEs need to be reported until week 20 of the study within the Netherlands.

9.5 Safety committee

For this study, a safety monitoring committee (SMC) will be appointed which will be independent of the investigators and sponsor and has no conflict of interest with the sponsor of the study. This SMC will consist of three experienced clinicians qualified to evaluate safety data from clinical studies with schistosome infections. Their main responsibility will be assessing safety reports or serious adverse events and advising the sponsor/investigator on trial continuation. Dose will be escalated 10 weeks after infection, i.e. 2 weeks after treatment, of a dose group, whereby <100% of volunteers were infected (showed detectable serum CAA). This can be a group of 3 or a group of 10 volunteers. The SMC can advise the sponsor of the trial not to escalate the dose, stop the trial, withdraw individual subjects or suspend further study procedures. Should the sponsor decide not to fully implement the advice of the SMC, the sponsor will send the advice to the reviewing METC, including a note to substantiate why (part of) the advice of the SMC will not be followed.

10. STATISTICAL ANALYSIS

10.1 Primary study parameter(s)

10.1.1 Frequency and severity of adverse events

All volunteers exposed to female *Sm* cercariae will be included in the intention to treat analysis. The safety and tolerability of controlled infection with female *Sm* is evaluated by tabulating all adverse events for each volunteer in an intention to treat analysis. Adverse events will be analysed by calculating the proportion of volunteers in each group who reported mild, moderate or severe adverse events. Given the very small group size, adverse events will mostly be descriptive. If relevant, statistical testing will be performed using chi-square tests or Fisher's exact tests.

10.1.2 Attack rate per dose group

The attack rate will be calculated by division of the number of volunteers with female *Sm* infection by the total number of volunteers in the per protocol analysis, multiplied by 100% (e.g. POS/TOTAL subjects * 100%). Volunteers withdrawing from the trial procedures at any time after infection and before the end of the study, will be tabulated as positive and included in the per protocol analysis if they had a serum CAA ≥1 pg/mL at any timepoint after exposure and before praziquantel treatment. They will be excluded from the analysis if there was no positive serum CAA sample until the moment of withdrawal.

10.2 Other study parameters

Delays in the time to positive serum or urine CAA test will be examined using Kaplan-Meier plot and log-rank (Mantel Cox) test, whereby different dose groups or a historical comparison with the male *Sm* cercariae infection will be made.
In the exploratory immunological analyses we will assess differences by comparing mean values between the dose groups or the infected/uninfected individuals using either a two-tailed student's t-test or non-parametric equivalents, paired if pre-infection values are compared with post-infection values, unpaired if comparisons are made between independent groups. For categorical variables (e.g. the number of positive assays), the chi-squared test or Fisher's exact test will be used (two-tailed). Similar to the CAA kinetics, historical comparison with the male *Sm* cercariae infection will be made.
To compare the metabolic changes after infection targeted metabolomics and changes in the microbiome, a straightforward multivariate analysis with a more general screen and multivariate modeling with principal component analysis (PCA) and partial least square

(PLS) analysis will be used. Also specific approaches for the time-resolved analysis will be used, multilevel analysis and/or (ANOVA)-simultaneous component analysis (ASCA).

11. ETHICAL CONSIDERATIONS

11.1 Regulation statement

The study will be conducted according to the principles of the Declaration of Helsinki (amended by 64th WMA assembly, Fortaleza, Brazil, published JAMA November 27, 2013 Volume 310, Number 20) and in accordance with the Medical Research Involving Human Subjects Act (WMO)

11.2 Recruitment and consent

Following approval by the accredited METC, advertisements will be placed in prominent places in public places as well as social media, on the website of the LUMC department of parasitology and infectious diseases and www.vaccinonderzoek.nl. Furthermore, a Facebook page showing the advertisement text will be designed to inform people about the trial and advertisements will be placed on local student websites.

The investigators will be responsible for providing adequate verbal and written information regarding the objectives and procedures of the study, the potential risks involved and the obligations of the volunteers. Volunteers will be informed that they will not gain health benefits from this study. Trainees or other students who might be dependent on the investigators or the study group will not be included in the study. After free discussion with the investigator, the volunteer will be given sufficient time, at least 72 hours, to consider participation. During the screening visit written informed consent is obtained from the volunteer. A sample volunteer information letter containing this information and informed consent form can be found in Sections E1 and E2.

Volunteers will be informed that they can withdraw their informed consent at any time during the study.

11.3 Benefits and risks assessment, group relatedness

There is no benefit of participation in the trial for the individual volunteer. Therefore the risk and discomfort of participation for individual volunteers should be carefully weighed against the scientific advantage of the controlled human *Schistosoma mansoni* infection model, in particular for vaccine and drug development. The CoHSI model has the potential to be extremely instrumental to vaccine and drug discovery and clinical testing for the widespread disease of *Schistosoma mansoni*, with an enormous global disease burden, and as such may have unprecedented repercussion for public health. Moreover,

the development of a female-only controlled human *Sm* infection model in addition to the previously established male-only model will allow further exploration of sex-specific immune responses.
The risks associated with participation in this study are related to infection with female helminths from the species *Schistosoma mansoni*. Several aspects should be considered in the risk assessment:

1. Risks of infection with female Schistosoma mansoni
   Please refer to section 2.3.2 of the Product Dossier for a description of the clinical experience with *Schistosoma mansoni* infections in travelers and the male controlled human *Schistosoma mansoni* infection.
   In summary, three main complications could occur following infection with female *Schistosoma mansoni* cercariae:
   a. Swimmer's itch
   A discrete urticarial rash at the site of entry of cercariae. The chances of this complication occurring is very likely, however, we expect the rash to be very mild as the number of cercariae penetrating the skin is low. In the CoHSI1 study 16 out of 17 volunteers reported a mild rash at the site of infection. All these rashes started within two days after the exposure and severity peaked one week after infection. None of the rashes required treatment. In case severe discomfort occurs, the urticarial rash can be treated with local triamcinolone cream 0.5%.
   b. Acute schistosomiasis syndrome
   A flu-like syndrome with protracted low to medium grade fever, fatigue, myalgia, malaise, non-productive cough, eosinophilia and patchy infiltrates on chest radiography can occur. Because it is unclear whether an acute schistosomiasis syndrome is related to the number of infected schistosomes or oviposition, it is hard to predict the chances of Katayama fever occurring and in which dose group. In the CoHSI1 study, two volunteers (out of three) in the 30 cercariae dose group developed symptoms of an acute schistosomiasis syndrome [11] that resolved without treatment. Another two (out of 11) in the 20 cercariae dose group developed severe AEs suggestive acute schistosomiasis syndrome [11]. In severe cases the syndrome can be treated symptomatically with analgesics, NSAIDs and corticosteroids.
   c. Morbidity related to oviposition
   Eggs trapped in the intestines or liver can induce a granulomatous host immune response that may present as abdominal pain, diarrhoea, and rectal

bleeding. The frequency of these symptoms is positively associated with the infection intensity. In severe cases, chronic inflammation may lead to periportal fibrosis resulting in clinical symptoms observed in individuals with portal hypertension, such as hepatomegaly, ascites, or oesophageal varices. The time between initial infection and these advanced stages are approximately 5-15 years [19]. In contrast to the male-only infections where egg production was never reported, we and others have in rare occasions observed egg-production in single-sex female infection of mice [20]. The numbers of eggs recovered were substantially lower as compared to mixed infections (please also see paragraph 13.1) and were never found to contain a viable miracidium. Possibly, the single female can produce and secrete egg shells on rare occasions. To reduce the possible production and number of egg(shells) we have adjusted the dose schedule to as low as possible doses of female cercariae and will initiate treatment as soon as the worm reaches adulthood and is possibly capable of producing eggs.

d. Acute toxemic neuroschistosomiasis

Neurological symptoms, such as low back pain irradiating to the limbs, lower limb muscle weakness, paresthesia, acute encephalitis, encephalomyelitis, aseptic meningitis are rare complications of schistosomiasis and thought to occur as a consequence of an inflammatory response of the host against the Schistosoma eggs deposited in the central nervous system by ectopic worms. Given the fact that this complication, even in endemic areas where worm burden is high, is rare, it is highly unlikely to happen after infection with female *Schistosoma mansoni* only.

2. Risks related to treatment with praziquantel

   Please refer to section 2.3.2.2. of the Product Dossier for an extensive description of the available treatment options. In summary, praziquantel is the only registered, extremely safe and highly effective drug for the treatment of *Schistosoma mansoni* infections. Because it is active on the mature worms only, treatment is recommended from 2-3 months after infection. Praziquantel is extremely safe with no long-term toxicity also for infants and pregnant women [6, 21, 22]. The short-term side effects are primarily fatigue, malaise, gastro-intestinal side effects and dizziness (see SPC Praziquantel) which are directly correlated with the pre-treatment intensity of infection [22]. Because of the risk of dizziness, volunteers are advised not to drive a car or operate machinery for the day of treatment and the day thereafter. In very rare instances, allergic reactions have been reported [22].

3. Possibility to abrogate infection
   Artemisinin derivatives are a good alternative option for the treatment of *Schistosoma mansoni,* and may be useful in the early phase of infection, as it is also active on immature stages. This drug may thus be used to abrogate early infections in case of withdrawal of study discontinuation. Artemether/lumefantrine will be given in an oral loading dose of 4 mg/kg, followed by 2 mg/kg for 3 consecutive days (same treatment regime as for malaria). Because artemether is not available as single drug in the Netherlands, it can only be supplied as a combination drug with lumefantrine (artemether/lumefantrine, AL). The most common side effects of artemether/lumefantrine are headache, dizziness, coughing, myalgia or arthralgia and gastrointestinal side effects. For a complete description of side effects, please refer to the “bijsluiter” (see SPC artemether/lumefantrine).

In summary, volunteers of the trial have a substantial risk of discomfort. However, side effects can be managed both symptomatically and with directed treatment at any time during the study.

11.4 Compensation for injury

The LUMC has a liability insurance which is in accordance with article 7 of the WMO. The LUMC (also) has an insurance which is in accordance with the legal requirements in the Netherlands (Article 7 WMO). This insurance provides cover for damage to research subjects through injury or death caused by the study. The insurance applies to the damage that becomes apparent during the study or within 4 years after the end of the study.

11.5 Incentives

Volunteers participating in the trial will receive €50,- per visit attended. If volunteers complete the trial, an additional €100,- will be awarded. The total compensation will therefore amount to €1100,-. We will pay out the initial €950,- after week 20 and the final €150,- after the 52 weeks follow-up visit. In case a volunteer misses a visit due to reasons other than force majeure the €50,- for this visit will not be awarded. Travel expenses will not be additionally reimbursed. In case of unexpected medical complications, there will be access to state-of-the-art medical treatment with full costs covered by the insurance of LUMC.

12. ADMINISTRATIVE ASPECTS, MONITORING AND PUBLICATION

12.1 Handling and storage of data and documents

All clinical trial data, serum samples, or other volunteer material will be labelled only with the volunteer study identification number. This is a unique code for each volunteer that is not relate to any personal identifiers. Only the investigators will have access to the key of the study ID's and to the names or birth year of the volunteers, which will be noted on a subject identification code list. This list will be kept in a locked cupboard by the Principal Investigator. Samples will be stored in our designated -80°C clinical trials freezers (located in C3-71 and S3-11) and in our liquid nitrogen tank (located in S3-11). Access to the rooms is restricted and door movements and temperature are logged. Personal information will not be disclosed to other members of the study team or third parties. Monitors from the LUMC monitor pool will have access to all source data during a monitoring visit. Medical confidentiality will be respected throughout the trial. Samples will be stored for at least 15 years. If a volunteer withdraws his consent, the coded data and samples collected till that moment will be used for analyses. Contact information of the LUMC data protection office can be found on page 4 of this protocol.

12.2 Monitoring and Quality Assurance

Monitoring will be executed by (internal) monitors of the LUMC according to the monitor plan. During the study, the data monitor will visit the site to check the completeness of patient subject records, the accuracy of entries on the eCRFs, the adherence to the protocol and to Good Clinical Practice.

The investigator will maintain source documents for each subject in the study. The investigator must also keep the original informed consent form signed by the subject (a signed copy is given to the subject).

The investigator will give the monitor access to all relevant source documents to confirm their consistency with the eCRF entries. The monitor will perform full verification for the presence of informed consent, adherence to the inclusion/exclusion criteria and documentation of SAEs. The recording of data that will be used for all primary and safety variables will be assessed for 100% of included subjects.

12.3 Amendments

A 'substantial amendment' is defined as an amendment to the terms of the METC application, or to the protocol or any other supporting documentation, that is likely to affect to a significant degree:

- the safety or physical or mental integrity of the subjects of the trial;

- the scientific value of the trial;
- the conduct or management of the trial; or
- the quality or safety of any intervention used in the trial.

All substantial amendments will be send to the METC for approval. The accredited METC will only be notified in case of non-substantial amendments.

12.4 Annual progress report

The investigator will submit a summary of the progress of the trial to the accredited METC once a year. Information will be provided on the date of inclusion of the first subject, numbers of subjects included and numbers of subjects that have completed the trial, serious adverse events/ serious adverse reactions, other problems, and amendments.

12.5 Temporary halt and (prematurely) end of study report

The sponsor will notify the accredited METC and the competent authority of the end of the study within a period of 90 days. The end of the study is defined as the last patient's last visit. The sponsor will notify the METC immediately of a temporary halt of the study, including the reason of such an action. In case the study is ended prematurely, the sponsor will notify the accredited METC and the competent authority within 15 days, including the reasons for the premature termination. Within one year after the end of the study, the investigator/sponsor will submit a final study report with the results of the study, including any publications/abstracts of the study, to the accredited METC and the Competent Authority.

12.6 Public disclosure and publication policy

The results of this trial will be published in a peer-reviewed journal unreservedly. Both negative and positive results will be published. Authorship arrangements will be made based on contribution on the report. This trial will be registered in a public trial registry.

13. STRUCTURED RISK ANALYSIS

13.1 Potential issues of concern

a. Level of knowledge about mechanism of action

Worldwide 200 million people are infected with Schistosome worms, of which *Schistosoma mansoni* is the main species. The pathophysiology of Schistosome infections have been described in detail (reviewed in [21]). However, natural infections differ from the proposed study in that bisexual infections occur in nature whereas we propose a controlled infection with female cercariae only. The main pathology induced by *Schistosoma mansoni* in endemic populations is caused by the excretion of eggs which may get trapped in tissues and provoke inflammatory, granulomatous and fibrous reactions. Severe disease develops in people with heavy long-standing infections [21]. In this study, we reduce the likelihood of egg-associated morbidity by only using female cercariae. In contrast with male worms, female adult schistosomes have been reported to rarely produce eggs, which have not been shown to contain a viable miracidium [23]. In a recent experiment at the LUMC, eggs were found in 1/6 (17%) female-only infected mice. In this particular mouse 2 eggs were recovered from the liver after 12 weeks of infection with 38 cercariae. The total number of eggs found in this mouse was much lower compared to the average number of eggs observed in mixed-sex infected mice (around 21,000). Microscopically the eggs appeared underdeveloped, a viable miracidium was not observed. Also in earlier reports eggs in female single sex infections were not viable [20]. Considering the proportionally large size of humans (compared to mice) and the low number of eggs, we estimate the risk of egg-associated morbidity in volunteers as a result of this study to be extremely small.

Also, people are repeatedly infected in the endemic situation whereas only one infection will occur in this controlled infection study. Thus the infection dose may be lower in the current study as opposed to natural infections. As such, the controlled infection model may be clinically best compared with the single dose infections which occur in travelers. In the male-only CoHSI study, we observed similar symptoms as those observed in travelers, namely swimmers' itch and acute schistosomiasis syndrome. For a detailed description please refer to the Product Dossier 2.3.2 Human exposure. Both syndromes may also occur after infection with single sex female cercariae.

Schistosoma mansoni worms reproduce sexually within the human host. A pair of male-female adult worms is able to produce hundreds of eggs per day [21]. However, worms do not multiply within the human host. Thus, a self-amplifying mechanism is highly unlikely. If an egg would accidentally be excreted into the environment through contaminated faeces, the life cycle *Schistosoma mansoni* would be unable to continue in the absence of the intermediate host, *Biomphalaria* snails, in The Netherlands, and adequate sanitation.

<u>b. Previous exposure of human beings with the test product(s) and/or products with a similar biological mechanism</u>

Controlled infections with female *Schistosoma mansoni* cercariae have not been performed in humans before, however we do have experience with controlled infection with male *Schistosoma mansoni* cercariae. We expect the symptoms related to the female worm maturation to be similar or less than those of the male worm maturation. This is supported by data from mice experiments that suggest the sex of cercariae differentially impacts host immune responses: infection with female cercariae appear to dampen host immune responses [12]. Consequently, symptoms associated with upregulated immune responses to worms, may be less apparent in female-only infections. In contrast with the male-only infection, in the female-only infection there is a small risk of egg-related symptoms. Please refer to paragraph 11.3 or the Product Dossier section 2.3.2 :Human exposure" for a detailed description of clinical syndromes associated with *Schistosoma mansoni* infection. In brief, the percutaneous penetration of cercariae may provoke a temporary urticarial rash which is usually discreet, limited to the site of entry and rarely requires medical attention [6]. Acute schistosomiasis syndrome (Katayama fever) is a systemic hypersensitivity reaction against the migrating schistosomulae, *Schistosoma* eggs or both, which occurs three to ten weeks after infection [6, 7, 21, 24-26]. The disease presents with fever, fatigue, myalgia, malaise, non-productive cough, eosinophilia and patchy infiltrates on chest radiography [21, 24-26]. Most patients recover spontaneously after 2-10 weeks. In our earlier study, acute schistosomiasis syndrome was more frequently observed in the higher dose group (30 cercariae) suggesting dose-dependency [11].

Abdominal symptoms, weight loss and hepatosplenomegaly has been described to develop at a later timepoint, which is caused by migration, positioning and oviposition of mature worms [21, 24, 25]. These symptoms are highly unlikely to occur, because worms do not mate and do not reach full sexual maturation. The risk of egg accumulation is low due to the single-sex nature of this study and treatment just after larval maturation.

Similarly, symptoms associated with chronic infection with *Schistosoma mansoni* are unlikely to occur because:

1. chronic infection pathophysiology is caused by egg deposition and its severity is linked to the infection intensity; although sporadically eggs are found in a female-only infection, the numbers are extremely low [6, 21];
2. the controlled infections are abrogated by curative treatment after 8 weeks of infection at which point oviposition may occur, making the duration of possible egg secretion by an adult female worm as short as possible.

c. Can the primary or secondary mechanism be induced in animals and/or in *ex-vivo* human cell material?

In vitro assays cannot capture the complexity of the life cycle of *Schistosoma mansoni* and the complex interaction with its host. Hamsters, mice and non-human primates (baboons) are susceptible species for *Schistosoma mansoni*, rodents are not able to harbour long-term chronic infections [27, 28]. As such, the pathophysiology of infections is best replicated in primates. However, infections in baboons are generally performed with a much higher dose of cercariae (600-1000 cercariae). A local urticarial rash has not been described in baboons after exposure. Rather, the pathological hallmark of schistosomiasis in baboons is the host anti-egg granulomatous response [9, 28]. A distinct Katayama fever syndrome, occurring before the gastrointestinal phase associated with oviposition has not been described.
As a result, extrapolation of pathophysiological and immunological aspects of infection to human disease is only possible to a very limited extent.

d. Selectivity of the mechanism to target tissue in animals and/or human beings

As a consequence of the co-evolution of *Schistosoma mansoni* with its human host, the effects that may be seen after controlled infection with female *Sm* cercariae are very likely specific to human tissue. As such, in vitro or animal models may not adequately mimic these effects.

e. Analysis of potential effect

Because the number of cercariae reaching a mature worm stage varies between 5-70%, we chose a starting dose of 10 cercariae to ensure development of at least one worm in the majority of volunteers. However, the occurrence of adverse events, particularly acute schistosomiasis syndrome, may not be dose related. Thus, close clinical follow-up is necessary to detect possible symptoms associated to infection. To this end, we scheduled weekly clinical trial visits and 24/7 availability of a trial physician. Adverse events will be recorded continuously. Should symptoms occur, additional diagnostics such as eosinophils and serum CAA tests may be performed to guide the clinical decision to treat

symptomatically or causally. The serum CAA test using up-converting phosphor labels is the most sensitive test for detection of antigen currently available [29].

f. Pharmacokinetic considerations

Not applicable

g. Study population

All subjects are healthy young adult volunteers, who have been screened for any evidence of co-morbidity. Female subjects of child-bearing age are screened for pregnancy by urine test and are required to use adequate contraception throughout the study period.

h. Interaction with other products

The primary drug for treatment of *Schistosoma mansoni* infections, praziquantel is metabolized through cytochrome P450, CYP3A4. Any inducers or inhibitors of this enzyme can thus interfere with the efficacy of praziquantel. Use of these drugs is an exclusion criterium for participation in the study.

Because praziquantel only acts on mature schistosomes, artemisinin derivatives may be used to abrogate early infections. In the Netherlands artemether/lumefantrine (AL) is the only available oral drug with an artemisinine derivative. Because lumefantrine may cause extension of QT-time, chronic use of drugs with effect on QT interval are excluded from the study. AL is metabolized through CYP3A4, but may affect drug levels in CYP2D6 metabolism. Chronic use of this drug is thus also excluded.

i. Predictability of effect

We expect that adverse effects of the infection will be observed in nearly all of the volunteers. Most volunteers will experience cercarial dermatitis at the site of infection (16 out of 17 in our previous trial with male cercariae). This is expected to resolve without intervention and none of the volunteers in the previous trial needed symptomatic treatment. Higher doses appeared to prolong the duration of the rash. Next, symptoms related to acute schistosomiasis infection are likely to occur in volunteers. In our previous study, there appeared to be some suggestion of higher risks with higher infection doses, however due to small sample size, statistical evidence for a dose-response relationship was difficult to assess. Because female worms are hypothesised to dampen innate host immune responses, the incidence of symptoms of acute schistosomiasis syndrome may be smaller in this study. The risk of morbidity related to oviposition is low, because the number of eggs secreted will be much lower than in a natural, mixed infection. In addition,

egg-related morbidity is positively associated with infection intensity and duration of infection. Our infection will be of low intensity (potentially similar to those observed in travelers) and of limited duration (8 weeks). The risk of side effects of praziquantel treatment increases with infection intensity, and therefore this risk is relatively low in our study.

The presence of *Schistosoma* worms can be detected with the UCP-CAA test. This test is the most sensitive and quantitative assay available to detect schistosome antigen. In baboons, we were able to show proof of concept by detecting low numbers of Schistosoma worms (figure 12, Product Dossier). However, the occurrence of adverse events (particularly acute schistosomiasis syndrome) may not be related to the level of antigen detected. Therefore, antigen levels are in principle only determined retrospectively and close clinical monitoring is required to ensure optimal safety and timely treatment of symptomatic volunteers.

j. Can effects be managed?

In case treatment would be required for dermatitis, triamcinoloncream 0.5% can be applied according to local clinical practice [13]. Acute schistosomiasis syndrome may be treated symptomatically with analgesics, non-steroidal anti-inflammatory drugs or corticoids [6, 21, 26]. The parasite load may be effectively reduced by over 90% after a single dose of 40 mg/kg praziquantel [6]. In our study, we treat the volunteers with two rounds of praziquantel 60 mg/kg at week 8 and week 12. The first dose is timed just after maturation of the larvae to further reduce any risk of egg accumulation. Some data suggests that the EC_{50} for single-sex female infection both in vivo (rodent) and in vitro [18] are higher than for mixed-sex infection indicating that a 60 mg/kg dose may be more appropriate. Validated sensitivity tests for *Schistosoma mansoni* are not available, but there is no firm clinical or epidemiological evidence for the emergence of praziquantel resistance in the field [21, 22, 30]. As an alternative to praziquantel, which is only active on the mature Schistosoma worm (~7 weeks post infection) [18, 22], artemisinin derivatives may be used to abrogate early infections [31]).

13.2 Synthesis

There is no benefit expected for volunteers participating in this study. The risk for volunteers after the controlled infection will be minimized by starting with very low dose cercariae and close observation of the volunteers. Strict inclusion and exclusion criteria have been implemented to exclude any comorbidities. The risks involved of participating in this study are the risk of swimmer's itch at the site of entry and acute schistosomiasis

syndrome several weeks after infection. To avoid chronic infection, all volunteers in the study will be treated with praziquantel twice. Because we can detect very low antigen levels in serum, we can repeat treatment until antigen is no longer detectable. Praziquantel is the only and first-line treatment for schistosomiasis. Praziquantel is generally well-tolerated, and the standard recommended dose is 40-60 mg/kg at one day. Artemisinine derivates are an excellent alternative treatment option in case infection needs to be abrogated in an early phase (<8 weeks after infection). Researchers at the LUMC have extensive experience with treatment of schistosomiasis in travelers. The SMC will be consulted to review all safety data before escalating the dose, to ensure thorough and independent expert opinion review of the data before continuing the trial. In conclusion, several measures have been put in place to ensure optimal safety of the volunteers and allow volunteers and investigators to abrogate the infection at any time. Based on clinical experience with *Schistosoma mansoni* infections in travelers and the previous controlled human infection study with male *Schistosoma mansoni*, the risks of participation in this trial are considered low. We thus believe that the scientific benefit will outweigh the risks of a controlled human female *Sm* infection model.

14. REFERENCES

1. *Global, regional, and national incidence, prevalence, and years lived with disability for 301 acute and chronic diseases and injuries in 188 countries, 1990-2013: a systematic analysis for the Global Burden of Disease Study 2013.* Lancet, 2015. **386**(9995): p. 743-800.
2. Hotez, P.J., et al., *The global burden of disease study 2010: interpretation and implications for the neglected tropical diseases.* PLoS Negl Trop Dis, 2014. **8**(7): p. e2865.
3. Mbabazi, P.S., et al., *Examining the relationship between urogenital schistosomiasis and HIV infection.* PLoS Negl Trop Dis, 2011. **5**(12): p. e1396.
4. Tukahebwa, E.M., et al., *Comparative efficacy of one versus two doses of praziquantel on cure rate of Schistosoma mansoni infection and re-infection in Mayuge District, Uganda.* Trans R Soc Trop Med Hyg, 2013. **107**(6): p. 397-404.
5. King, C.H., et al., *Utility of repeated praziquantel dosing in the treatment of schistosomiasis in high-risk communities in Africa: a systematic review.* PLoS Negl Trop Dis, 2011. **5**(9): p. e1321.
6. Clerinx, J. and A. Van Gompel, *Schistosomiasis in travellers and migrants.* Travel Med Infect Dis, 2011. **9**(1): p. 6-24.
7. Knopp, S., et al., *Diagnosis and treatment of schistosomiasis in children in the era of intensified control.* Expert Rev Anti Infect Ther, 2013. **11**(11): p. 1237-58.
8. Richter, D., D.A. Harn, and F.R. Matuschka, *The irradiated cercariae vaccine model: looking on the bright side of radiation.* Parasitol Today, 1995. **11**(8): p. 288-93.
9. Kariuki, T.M., et al., *Previous or ongoing schistosome infections do not compromise the efficacy of the attenuated cercaria vaccine.* Infect Immun, 2006. **74**(7): p. 3979-86.
10. Darton, T.C., et al., *Design, recruitment, and microbiological considerations in human challenge studies.* Lancet Infect Dis, 2015. **15**(7): p. 840-51.
11. Langenberg, M.C.C., et al., *A controlled human Schistosoma mansoni infection model to advance novel drugs, vaccines and diagnostics.* Nat Med, 2020.
12. Sombetzki, M., et al., *Host Defense Versus Immunosuppression: Unisexual Infection With Male or Female Schistosoma mansoni Differentially Impacts the Immune Response Against Invading Cercariae.* Front Immunol, 2018. **9**: p. 861.
13. Lupi, O., et al., *Mucocutaneous manifestations of helminth infections: Trematodes and cestodes.* J Am Acad Dermatol, 2015. **73**(6): p. 947-57; quiz 957-8.
14. Helleberg, M. and S. Thybo, *High rate of failure in treatment of imported schistosomiasis.* J Travel Med, 2010. **17**(2): p. 94-9.
15. WHO. *WHO Model Formulary, 2008. Based on the 15th Model List of Essential Medicines 2007*. [cited 2020 09-01]; Available from: http://apps.who.int/medicinedocs/documents/s16879e/s16879e.pdf.
16. Castro, N., et al., *Bioavailability of praziquantel increases with concomitant administration of food.* Antimicrob Agents Chemother, 2000. **44**(10): p. 2903-4.
17. Zwang, J. and P.L. Olliaro, *Clinical efficacy and tolerability of praziquantel for intestinal and urinary schistosomiasis-a meta-analysis of comparative and non-comparative clinical trials.* PLoS Negl Trop Dis, 2014. **8**(11): p. e3286.
18. Pica-Mattoccia, L. and D. Cioli, *Sex- and stage-related sensitivity of Schistosoma mansoni to in vivo and in vitro praziquantel treatment.* Int J Parasitol, 2004. **34**(4): p. 527-33.
19. Colley, D.G., et al., *A five-country evaluation of a point-of-care circulating cathodic antigen urine assay for the prevalence of Schistosoma mansoni.* Am J Trop Med Hyg, 2013. **88**(3): p. 426-32.
20. Shaw, M.K., *Schistosoma mansoni: vitelline gland development in females from single sex infections.* J Helminthol, 1987. **61**(3): p. 253-9.
21. Gryseels, B., *Schistosomiasis.* Infect Dis Clin North Am, 2012. **26**(2): p. 383-97.
22. Cioli, D., et al., *Schistosomiasis control: praziquantel forever?* Mol Biochem Parasitol, 2014. **195**(1): p. 23-9.
23. Waisberg, M., et al., *Microarray analysis of gene expression induced by sexual contact in Schistosoma mansoni.* BMC Genomics, 2007. **8**: p. 181.

24. Gryseels, B., et al., *Human schistosomiasis.* Lancet, 2006. **368**(9541): p. 1106-18.
25. Ross, A.G., et al., *Katayama syndrome.* Lancet Infect Dis, 2007. **7**(3): p. 218-24.
26. Pavlin, B.I., P. Kozarsky, and M.S. Cetron, *Acute pulmonary schistosomiasis in travelers: case report and review of the literature.* Travel Med Infect Dis, 2012. **10**(5-6): p. 209-19.
27. Warren, K.S. and E.G. Berry, *Induction of hepatosplenic disease by single pairs of the Philippine, Formosan, Japanese, and Chinese strains of Schistosoma japonicum.* J Infect Dis, 1972. **126**(5): p. 482-91.
28. Nyindo, M. and I.O. Farah, *The baboon as a non-human primate model of human schistosome infection.* Parasitol Today, 1999. **15**(12): p. 478-82.
29. van Dam, G.J., et al., *A robust dry reagent lateral flow assay for diagnosis of active schistosomiasis by detection of Schistosoma circulating anodic antigen.* Exp Parasitol, 2013. **135**(2): p. 274-82.
30. Ismail, M., et al., *Resistance to praziquantel: direct evidence from Schistosoma mansoni isolated from Egyptian villagers.* Am J Trop Med Hyg, 1999. **60**(6): p. 932-5.
31. Perez del Villar, L., et al., *Systematic review and meta-analysis of artemisinin based therapies for the treatment and prevention of schistosomiasis.* PLoS One, 2012. **7**(9): p. e45867.
